# A tutorial on causal network simulation and exploration using the causalnet R package

**DOI:** 10.3758/s13428-026-03030-z

**Published:** 2026-06-22

**Authors:** Kyuri Park, Vítor V. Vasconcelos, Mike Lees

**Affiliations:** 1 Computational Science Lab, Informatics Institute, University of Amsterdam, PO Box 94323, Amsterdam, 1090GH The Netherlands; 2 Institute for Advanced Study, University of Amsterdam, Oude Turfmarkt 147, Amsterdam, 1012GC The Netherlands

**Keywords:** Causal network, Network enumeration, Symptom dynamics, Psychological modeling

## Abstract

Understanding how network structure influences system dynamics is essential for advancing psychological modeling. This tutorial introduces the causalnet R package, which enables researchers to systematically enumerate candidate directed networks by orienting a user-specified undirected or partially directed adjacency template. Users can impose directional constraints—such as those derived from prior theory or time-series models (e.g., graphical vector autoregressive models)—to restrict the space of admissible directed network configurations. The package supports dynamic simulations on these networks using either a theoretically grounded nonlinear model (Park et al., 2026b) or a simplified linear alternative. Researchers can simulate system behavior and compare dynamic outcomes across structural configurations, parameter sets, or modeling assumptions. The primary audience is applied psychological and behavioral scientists who wish to evaluate competing theoretical accounts of symptom and behavior dynamics when causal direction is uncertain. Importantly, causalnet is not intended to identify a unique causal network from cross-sectional data; instead, it supports theory- and evidence-constrained enumeration of candidate directed structures and simulation-based screening of their dynamic implications against empirical targets. We illustrate how this workflow can be used to adjudicate competing psychological theories by linking structural assumptions to predicted dynamic signatures such as persistence and recovery. This approach facilitates a systematic exploration of how causal architecture and interaction dynamics give rise to the emerging dynamics of psychological processes over time.

**Supplementary Information:**

The online version contains supplementary material available at https://doi.org/10.3758/s13428-026-03030-z.

## Introduction

The network perspective on psychological phenomena has reshaped how researchers conceptualize the structure and dynamics of mental disorders, cognitive processes, and affective states. In this perspective, variables such as symptoms or behaviors are modeled as nodes in a dynamic system, with edges capturing statistical dependencies or causal influences among them (Borsboom & Cramer, 2013). Partial correlation networks are a popular starting point: estimated from cross-sectional data using tools such as the qgraph or bootnet packages, they represent undirected edges between nodes that are conditionally dependent (Epskamp et al., 2018a, b; Epskamp & Fried, 2025).

While such networks are valuable for descriptive and exploratory analyses, their undirected edges limit causal or temporal interpretation. When intensive longitudinal data are available, models such as the graphical vector autoregressive (GVAR) model (Epskamp, 2020) can estimate directed lagged relations. In many applied settings, however, researchers either lack time-series data or have uncertainty about directionality that is only partially resolved by available evidence. More broadly, both cross-sectional and time-series approaches often leave open the question of how *alternative* candidate causal structures would change predicted system dynamics.

Recent work highlights the importance of feedback loops—cycles of directed influence—in shaping the persistence, escalation, or recovery of psychological states. For example, Park et al. (2026a) show that networks with concentrated feedback loops tend to maintain elevated symptom levels after stress, slowing recovery. These findings suggest that it is not just the presence of edges, but how they are configured into cycles and overlapping pathways, that governs system behavior. Yet systematically exploring alternative directed structures and simulating their dynamic consequences can be computationally demanding and technically beyond the reach of many applied researchers. This problem is exacerbated by the fact that existing tools offer limited support for systematically analyzing or simulating such dynamics.

To address this gap and build on workflows familiar to psychological scientists, we introduce the causalnet R package. The package enables users to: (i) enumerate candidate directed networks by orienting a given undirected or partially directed skeleton; (ii) encode substantive assumptions as constraints (e.g., required, forbidden, or oriented relations); (iii) quantify feedback architecture using loop-based structural metrics; and (iv) simulate system dynamics on candidate network configurations using the default nonlinear model, a linear alternative, or a user-specified custom process model.

This tutorial provides a step-by-step walkthrough of the applied workflow supported by causalnet, linking substantive constraints to admissible directed structures and their predicted dynamics. Each step is first illustrated using a didactic three-node example and then demonstrated in an empirical application using symptom network data (Schramm et al., 2017). Throughout, we use the term “admissible” to mean “consistent with the skeleton and constraints”, not “most likely to be true.” Depending on the research question, researchers may compare simulated dynamics to qualitative or quantitative empirical benchmarks (e.g., baseline prevalence, co-activation patterns, autocorrelation, recovery dynamics, or intervention responses) to evaluate which structural assumptions are most compatible with observed phenomena.

Importantly, causalnet is not a causal discovery method for cross-sectional data. Its purpose is to support theory-driven exploration and sensitivity analysis when directionality is uncertain: researchers specify a defensible relation set (skeleton), encode theory- or evidence-based directional constraints, and then examine how remaining structural uncertainty changes predicted dynamics under explicit process-model assumptions. In other words, the package helps researchers ask “what dynamics would follow if these structural assumptions were true?” rather than claiming to identify the single best causal graph from observational data.

When the starting skeleton is estimated from a cross-sectional conditional-dependence model, such as a partial-correlation or Ising network, its undirected edges indicate retained statistical dependencies rather than confirmed direct causal relations. Accordingly, orienting such a skeleton should be interpreted as generating a constrained set of candidate directed interaction models conditional on the chosen adjacency template, rather than as recovering the full set of causal graphs compatible with the underlying data-generating process.

## Define a network skeleton and generate directed networks

A *network skeleton* specifies which pairs of variables are connected but leaves the direction of those connections unspecified. In applied psychological work, the skeleton is often a defensible summary of which processes are plausibly related, while causal direction remains uncertain or only partially constrained by theory, longitudinal evidence, or experiments. causalnet is designed for this setting: it treats directionality as a structured uncertainty to be explored systematically under explicit constraints. Skeletons are typically derived from partial correlation networks estimated from cross-sectional data and represented as symmetric adjacency matrices (Epskamp et al., 2018a, b. In this tutorial, such a skeleton is treated as an adjacency template that specifies which variable pairs are retained for directional exploration. This does not imply that a partial-correlation network is identical to the skeleton of a causal graph, because an undirected conditional-dependence edge does not necessarily correspond to a direct causal relation between those same two variables.^1^

In some cases, researchers may incorporate partial directionality—derived from time-series models such as the graphical vector autoregressive (GVAR) model (Epskamp, 2020), or from theoretical assumptions— resulting in a partially directed skeleton.

Crucially, many distinct directed networks can be consistent with a single skeleton. For example, a fully connected triangle of three nodes yields 27 distinct directed graphs when both unidirectional and bidirectional edges are permitted. These alternatives may differ substantially in their causal interpretations, feedback loop structures, and implications for system dynamics. In applied settings, this enables theory-driven comparison of competing directional hypotheses (encoded as constraints) by examining which candidate directed structures yield psychologically plausible dynamic signatures (e.g., persistence vs. recovery after stress) and/or best match pre-specified empirical targets. However, most estimation methods return only a single network and do not account for this structural uncertainty.

To keep the tutorial concrete while remaining broadly applicable, we begin with a minimal three-node example (a fully connected triangle). The example is intentionally generic: it is used to illustrate the workflow for handling directional uncertainty (skeleton *→* candidate directed ensemble *→* structural summaries) without committing to a specific substantive domain. Readers may map the three nodes onto a simple conceptual system (e.g., three mutually related symptoms or processes), where theory suggests connections exist but does not uniquely determine all causal directions. This mirrors a common applied scenario: a researcher has a theoretically/empirically motivated skeleton of relations and partial directional expectations, but wishes to systematically explore alternative directed causal structures that remain consistent with the chosen skeleton and constraints. We use this example to demonstrate how causalnet enumerates possible edge orientations under a fixed skeleton, and how alternative directed structures can imply different feedback architectures and, consequently, different dynamic behavior. We represent the skeleton as a symmetric adjacency matrix and visualize it using the qgraph package:

**Figure:**
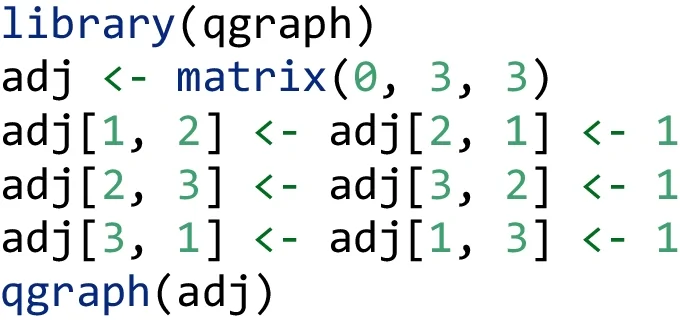

We then use the generate_directed_networks() function from the causalnet package to create an ensemble of networks that enumerates all valid directed networks compatible with the skeleton:

**Figure:**
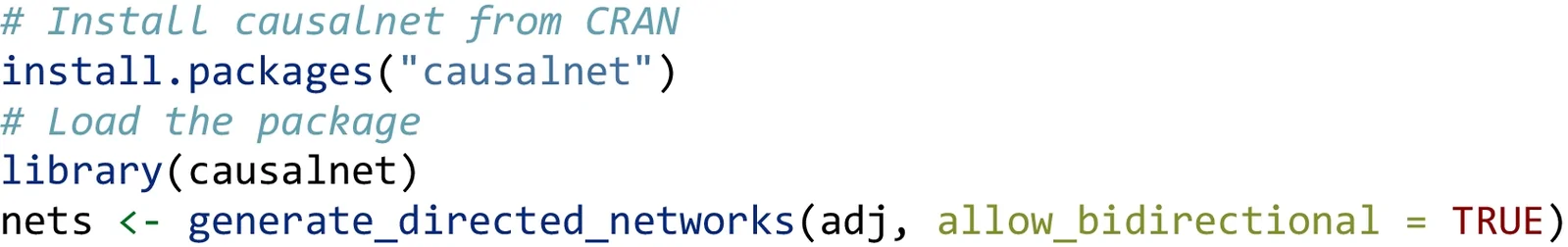

**Fig. 1:**
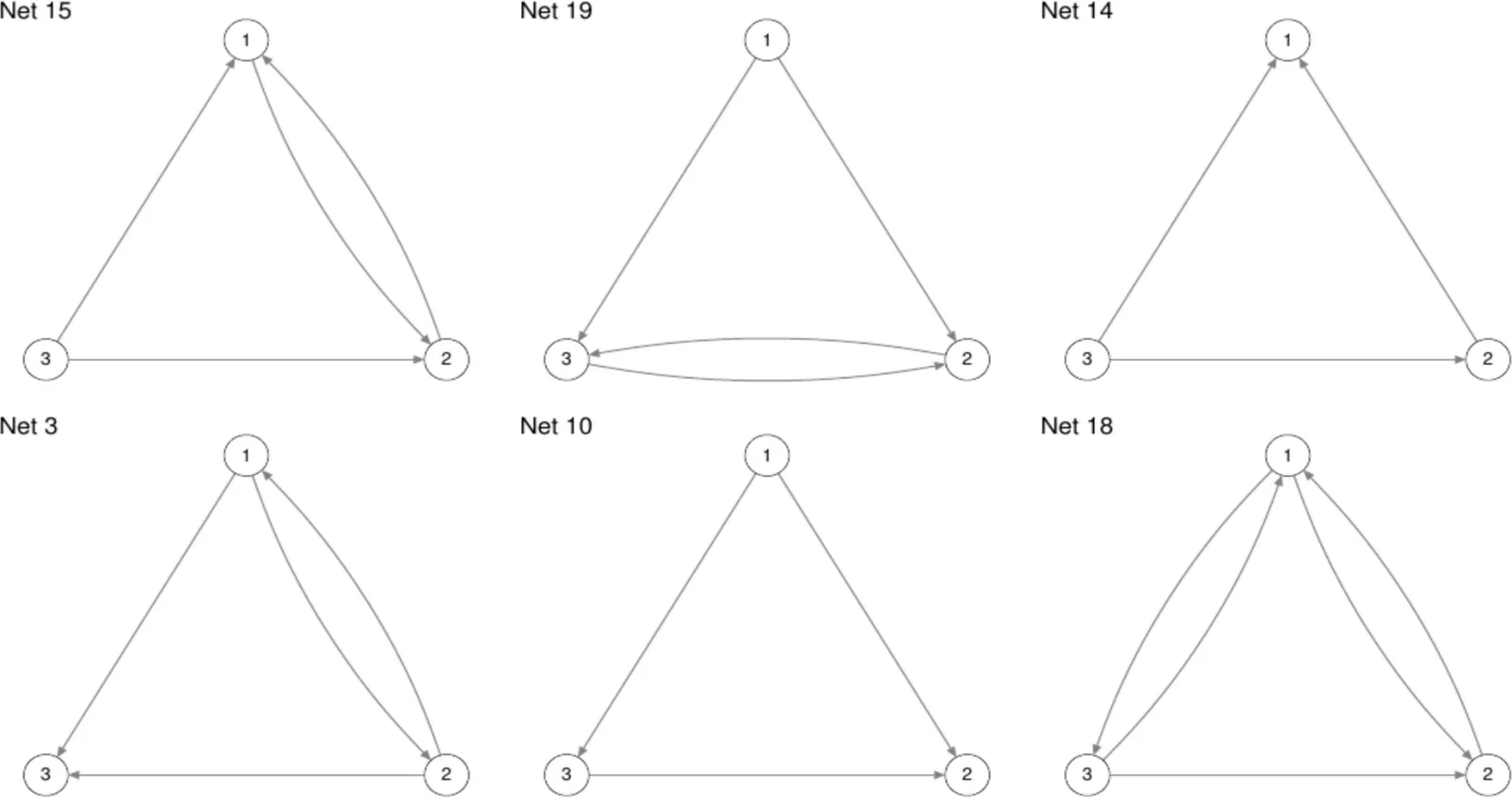
Random sample of six directed networks consistent with a three-node undirected triangle skeleton

In causalnet, the undirected skeleton is treated as an adjacency template whose retained edges are subsequently oriented. Thus, the enumeration step should be understood as generating candidate directed network configurations conditional on that template. Each undirected edge can be assigned one of three forms: A → B, A ← B, or A ↔ B. If the argument allow_bidirectional is set to FALSE then the third edge type is not possible, eliminating all dyadic loops from the set of possible networks. Users may also impose custom directional constraints via the fixed_edges matrix: an entry set to 1 fixes an edge as directional from A → B, 2 enforces bidirectionality A ↔ B, and NA (or 0) leaves the edge unconstrained. See help(generate_directed_networks) for a full list of available arguments and customization options. Note that fixed_edges constrains only the *orientation* of edges that are already present in the undirected skeleton adj_matrix. To rule out a direct relationship entirely, users should remove that edge at the skeleton stage by setting adj_matrix[i,j] = adj_matrix[j,i] = 0 before calling generate_directed_networks(). Figure 1 shows a random sample of six directed networks that are structurally consistent with this undirected triangle skeleton. The following code reproduces Fig. 1.

**Figure:**
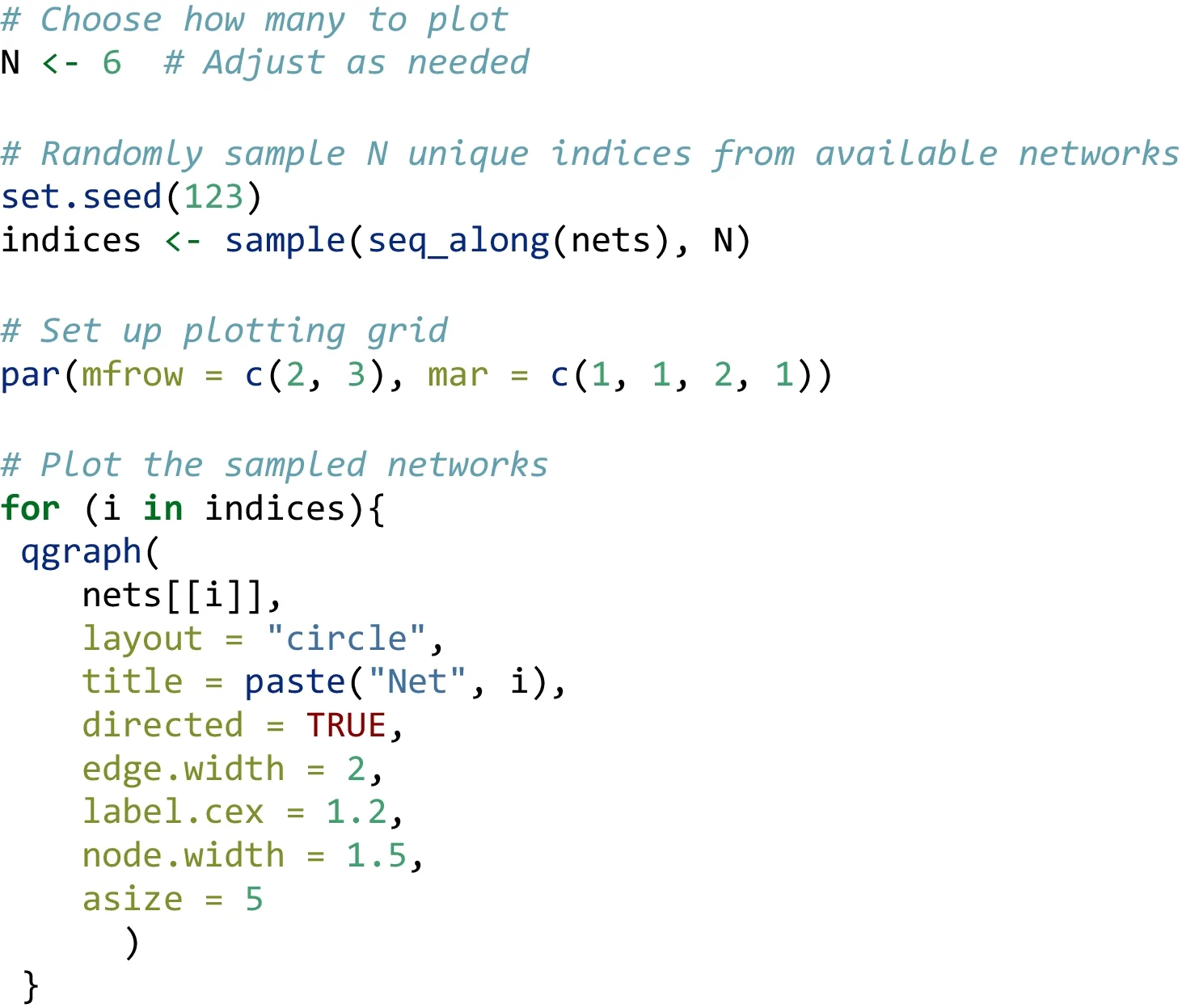

## Characterize causal architecture

For applied researchers, the key question is not only which directed structures are admissible under the skeleton and constraints, but how those structures differ in psychologically meaningful architecture—for example, whether feedback is abundant, localized, or concentrated around a small set of nodes. We therefore summarize each admissible directed network using loop-based metrics that make these architectural differences explicit. Given an ensemble of directed networks, the next step is to characterize their internal causal structure. Among various topological features, feedback loops and directed cycles through which activation can persist are of particular interest due to their potential role in symptom maintenance and recovery dynamics (Borsboom 2017; Park et al. 2026b; Isager 2023). The causalnet package provides tools to detect and summarize feedback architecture, alongside other structural properties relevant to psychological processes. To support systematic analysis of these features, causalnet includes the function summarize_network_metrics(). This function accepts a list of directed adjacency matrices (e.g., from generate_directed_networks()) and returns a data frame summarizing each network’s topological profile.

**Figure:**
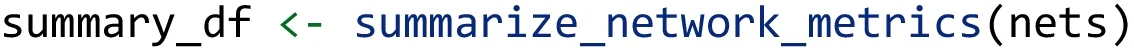

By default, the function returns four structural metrics, in addition to basic metadata variables such as net_id, n_nodes, and n_edges. The number of nodes is fixed across networks, whereas the number of edges may vary when bidirectional edges are allowed, depending on the number of such edges in each configuration. In practice, these metrics let researchers quickly screen an admissible ensemble for qualitatively different structural regimes (e.g., loop-sparse vs. loop-dense, diffuse vs. concentrated feedback), and then select representative candidates for simulation-based comparison.

- *Number of feedback loops* (num_loops): The total count of unique directed cycles in the network. Cycles that differ only in their starting point or traversal order (e.g., 1→2→3→1 vs. 2→3→1→2) are considered equivalent and counted once.
- *Degree variability* (sigma_total): This metric summarizes heterogeneity in node connectivity. Specifically, for each network, we compute the standard deviation of the incoming weighted degree distribution (${\sigma}_{in}$) and the standard deviation of the outgoing weighted degree distribution across nodes (${\sigma}_{out}$), and then sum these quantities (${\sigma}_{tot}$ = ${\sigma}_{in}$ + ${\sigma}_{out}$). Networks with hubs or asymmetric structure will have higher variability.
- *Node overlap score* (node_overlap_score): This quantifies how strongly feedback loops are concentrated around specific nodes. It is calculated as $Overlap Score=\frac{\sum_{i}freq{\left(i\right)}^{2}}{{L}^{2}}$, where $freq\left(i\right)$ is the number of detected loops that include node i and L denotes the total number of loops. Squaring $freq\left(i\right)$ upweights nodes that participate in many loops, so the score increases when many loops repeatedly involve the same subset of nodes (i.e., overlapping feedback organized around “loop hubs”). Conversely, lower values indicate that loop participation is more evenly distributed across nodes, with less reuse of the same nodes across loops. Because the score aggregates node-wise contributions ${\left(freq\left(i\right)/L\right)}^{2}$ across all nodes, its magnitude should be interpreted relative to network size (n). In practice, the overlap score is most informative when used comparatively (e.g., to contrast lower- versus higher-overlap candidate networks within the same ensemble generated from a given skeleton and set of constraints).
- *Average loop size* (avg_loop_size): This is the mean number of nodes involved in each feedback loop, computed by averaging the sizes of all unique directed cycles in a network. It provides an indication of how localized (small loops) or widespread (large loops) the feedback structures are. For networks without loops, this value is marked as *not available* (NA).

Taken together, these four metrics provide an “architecture profile” of each candidate network: how much feedback is present (num_loops), how large feedback cycles tend to be (avg_loop_size), and how feedback is distributed across nodes (sigma_total and node_overlap_score). The resulting summary data frame has one row per network and one column per metric. These features can be used for exploratory analysis, classification, or as predictors in structure–function modeling. To visualize the distribution of these metrics across networks, you can use the built-in plotting function plot_network_metrics:

**Figure:**
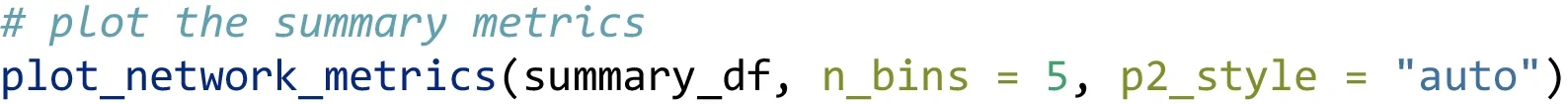

For panel (b) (degree variability by loop count), plot_network_metrics() supports multiple point-layout options. By default (p2_style = “auto”), the function uses a stacked/beeswarm layout for smaller ensembles (fewer than 200 networks by default) and switches to jitter for larger ensembles where stacking becomes overly dense. Users can override this behavior by setting p2_style to “point” (no jitter), “jitter”, or “beeswarm”.

**Fig. 2:**
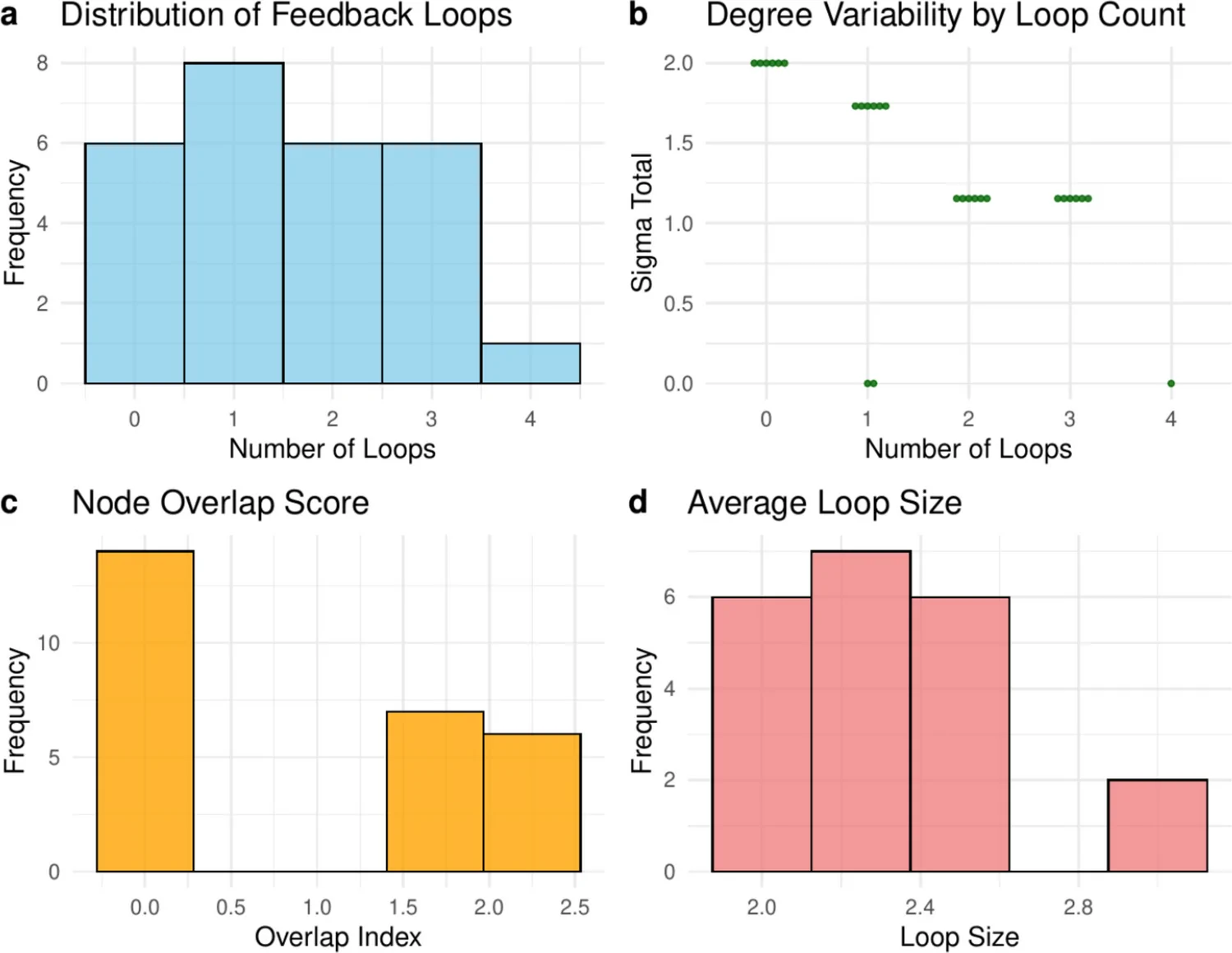
Summary of loop-based structural metrics across all directed networks generated from the triangle skeleton. Panels show the distribution of the number of feedback loops, degree variability, node overlap score, and average loop size

Figure 2 illustrates how these metrics vary across the 27 directed networks derived from a three-node triangle skeleton. Although each network is structurally consistent with the same undirected skeleton, their feedback architectures diverge. Some networks exhibit no loops at all, while others contain tightly overlapping cycles.

Together, these metrics provide a compact summary of each network set and its causal topology, and highlight structural features that may influence system behavior. For example, researchers can use them to identify candidate networks for dynamic analysis, prioritize loop-dense or loop-sparse configurations, or examine how specific topological traits—such as high node overlap—relate to persistence or reactivity in dynamical systems.

## Simulate system dynamics

In psychological applications, this step supports theory-driven questions such as whether a proposed causal architecture yields persistence versus recovery following a perturbation, or whether intervening on a candidate node produces qualitatively different trajectories. Having summarized structural variation across networks, we now turn to simulating system dynamics to examine how different causal architectures give rise to distinct patterns of behavior. The causalnet package includes both linear and nonlinear stochastic models for simulating dynamics, along with tools for parameter specification and visualization of system trajectories. Together, these functions allow users to explore how features such as feedback loops, external perturbations, and parameter uncertainty shape the evolution of system states over time.

## Model for simulating dynamics

Before turning to the practical aspects of simulation, we briefly outline the default model implemented in causalnet. This model is a nonlinear stochastic differential equation (SDE) introduced by (Park, Waldorp, and Vasconcelos 2026b), designed to capture key properties of psychological systems. It incorporates nonlinearity, self-excitation, feedback amplification, and bounded activation into a flexible dynamical framework.

## Model equation

For each node i, the corresponding state variable ${S}_{i}\left(t\right)$ evolves over time according to:

$$d{S}_{i}\left(t\right)={S}_{i}\left(1-{S}_{i}\right)\left[{\beta}_{i}+{\alpha}_{self,i}{S}_{i}\left(t\right)+\sum_{j}{A}_{ij}{S}_{j}\left(t\right)\left(1+{\delta}_{i}{S}_{i}\left(t\right)\right)\right]dt+{\sigma}_{i}\hspace{0.17em}d{W}_{i}\left(t\right)$$

where:

- ${A}_{ij}$ is an entry in the directed adjacency matrix, representing the strength and direction of influence from node j to node i (with ${A}_{ij}=0,$ indicating no connection).
- ${\beta}_{i}$ is a baseline input or activation bias,
- ${\alpha}_{self,i}$ controls self-excitation or self-regulation of node i,
- ${\delta}_{i}$ modulates the nonlinear amplification of incoming influence based on the current state ${S}_{i}$,
- ${\sigma}_{i}$ controls the intensity of random noise,
- $d{W}_{i}\left(t\right)$ is a Wiener process (i.e., standard Brownian motion), with reflective boundaries.

Logistic terms ${S}_{i}\left(1-{S}_{i}\right)$ and noise reflective boundaries ensure that the state values remain bounded within the interval $\left[0,1\right]$. This constraint is useful for modeling systems where variables have natural upper and lower limits, such as levels of engagement, stress, activation, or belief certainty. The multiplicative term involving ${\delta}_{i}$ introduces state-dependent gain, which means that the nodes become more sensitive to inputs as they become more activated. This dynamic captures cascading effects and nonlinear escalation patterns that can arise in a variety of feedback-driven systems and can be used for sensitivity to added nonlinearity.

Although the nonlinear model is the default in causalnet, users may also opt for a simpler linear model for exploratory purposes. The linear version omits both the logistic terms ${S}_{i}\left(1-{S}_{i}\right)$ and the nonlinear amplification term ${\delta}_{i}$, resulting in the following form:

$$d{S}_{i}\left(t\right)=\left[{\beta}_{i}+{\alpha}_{self,i}{S}_{i}\left(t\right)+\sum_{j}{A}_{ij}{S}_{j}\left(t\right)\right]dt+{\sigma}_{i}\hspace{0.17em}d{W}_{i}\left(t\right)$$

In this formulation, state values are not bounded and may grow without limit (i.e., exhibit runaway dynamics), particularly in feedback-rich networks, unless manually constrained. While the linear model does not support nonlinear behaviors such as bistability or hysteresis, it can still offer useful insights into the general direction and stability of system trajectories under simpler assumptions, and it also makes it easier to track how specific structural features influence dynamic behavior.

## Sampling parameters for simulation

To simulate dynamics on a given network, we must first define a set of node-level parameters that govern how each variable behaves over time. The get_sample_parameters() function in causalnet is designed to generate a complete set of these parameters, either by drawing random values from default ranges or by accepting user-specified values. This function returns a named list with one value per node for each of four key parameters:

- beta: Baseline activation bias, which controls how active a node tends to be in the absence of input. *Default*: Uniform (-1.5, -1).
- alphaSelf: Self-excitation, which captures how strongly a node reinforces its own state. *Default*: Uniform (0.05, 0.3).
- delta: Nonlinear amplification, which magnifies incoming influence when a node is already active. Used only in the nonlinear model. *Default*: Uniform (1, 5).
- sigma: Noise strength, this scales the impact of random fluctuations. *Default*: Uniform (0.01, 0.1).

Each parameter is returned as a numeric vector of length equal to the number of nodes. These are passed directly to simulation function simulate_dynamics().

A basic usage example:

**Figure:**
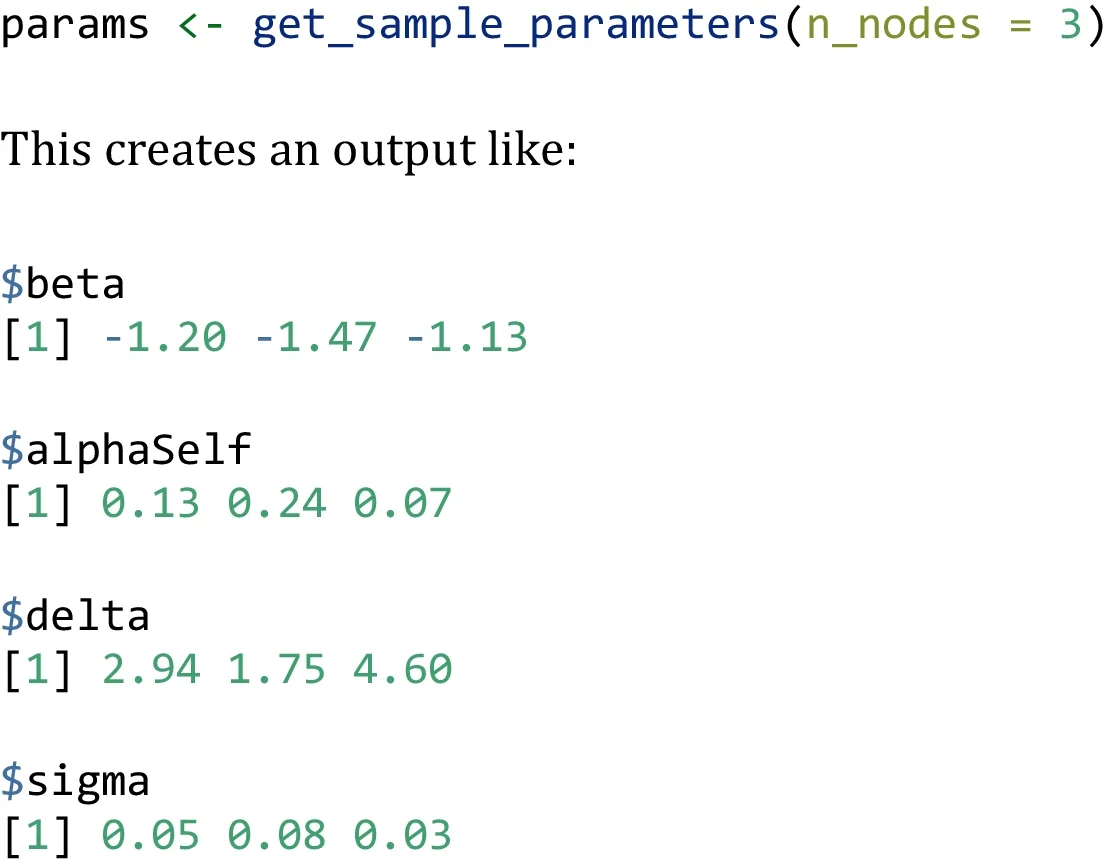

If the user wants full control, any of these parameters can be manually specified by supplying a numeric vector. For instance:

**Figure:**
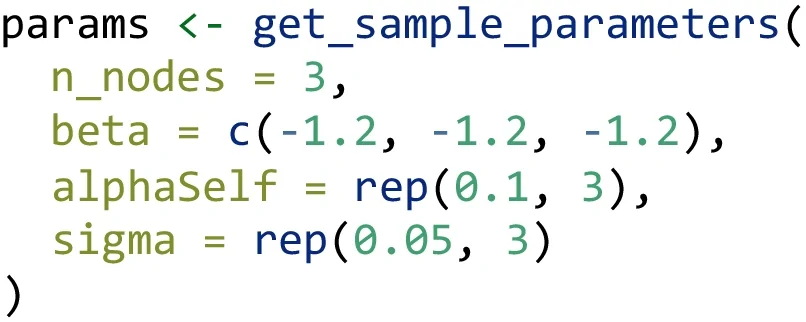

In this case, only the delta parameter is sampled, while the others remain fixed. This design enables reproducible and flexible simulations, allowing researchers to keep some parameters constant while systematically varying others. Such parameterization is essential for exploring how causal architecture interacts with dynamic processes. Even with the same network structure, small changes in baseline activation or feedback amplification can produce markedly different system behaviors (Isager 2023; Borsboom 2017; Cramer et al. 2016; Strogatz 2001). By deliberately varying the parameters, users can assess how robust their conclusions are to parametric uncertainty (Iooss & Lemaître, 2015; Saltelli et al., 2004, 2008).

## Run simulation

Once the parameters have been specified, the dynamics of the system can be simulated using the simulate_dynamics() function—the core engine for temporal modeling in causalnet. This function numerically integrates a stochastic differential equation (SDE) over time and supports three model types: the default nonlinear formulation, a simpler linear alternative, or a fully user-defined model. The nonlinear model is particularly suited for exploring persistence, escalation, and recovery in feedback-rich systems, especially when simulating bounded or dampened dynamics. Users can switch to the linear model by setting model_type = “linear”, or supply their own custom model using the model_fn argument, allowing flexible experimentation with alternative psychological processes. Details and worked examples of model_fn customization are provided in the Appendix.

To run a simulation, users must provide a directed adjacency matrix (adj_matrix), a list of node-level parameters (params), and basic simulation settings that include total duration (t_max) and timestep size (dt). Additional optional arguments include an initial state vector (S0, defaulting to 0.01 for all nodes), the model type (“nonlinear”, “linear”, or NULL for custom), a user-defined model function (model_fn), and an optional stress input function (stress_event) for simulating external perturbations during specific time windows.

The example below illustrates the minimal workflow: defining a temporary stressor (active between timepoints 10 and 15), sampling parameters for a three-node network, and running a simulation for 50 time units. Here, my_net denotes any directed adjacency matrix; for illustration, we use one of the enumerated networks generated in the previous step (e.g., my_net <- nets[[1]]). Complete, end-to-end examples that reproduce the manuscript figures are provided in the subsequent sections (“Comparing Dynamics: Loop-Free vs. Loop-Rich Networks” and “Applied Example: Modeling Symptom Networks”).

**Figure:**
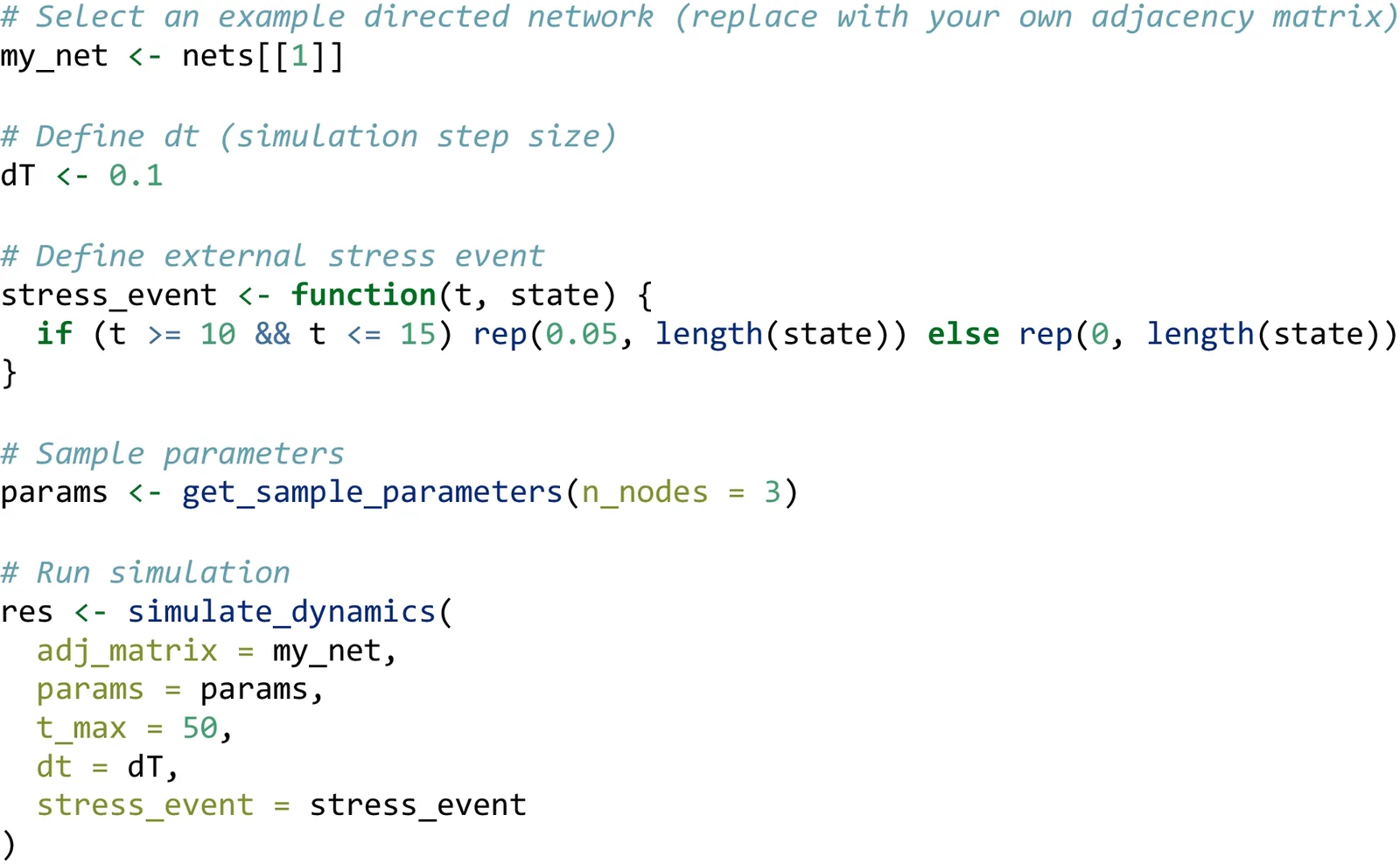

**Fig. 3:**
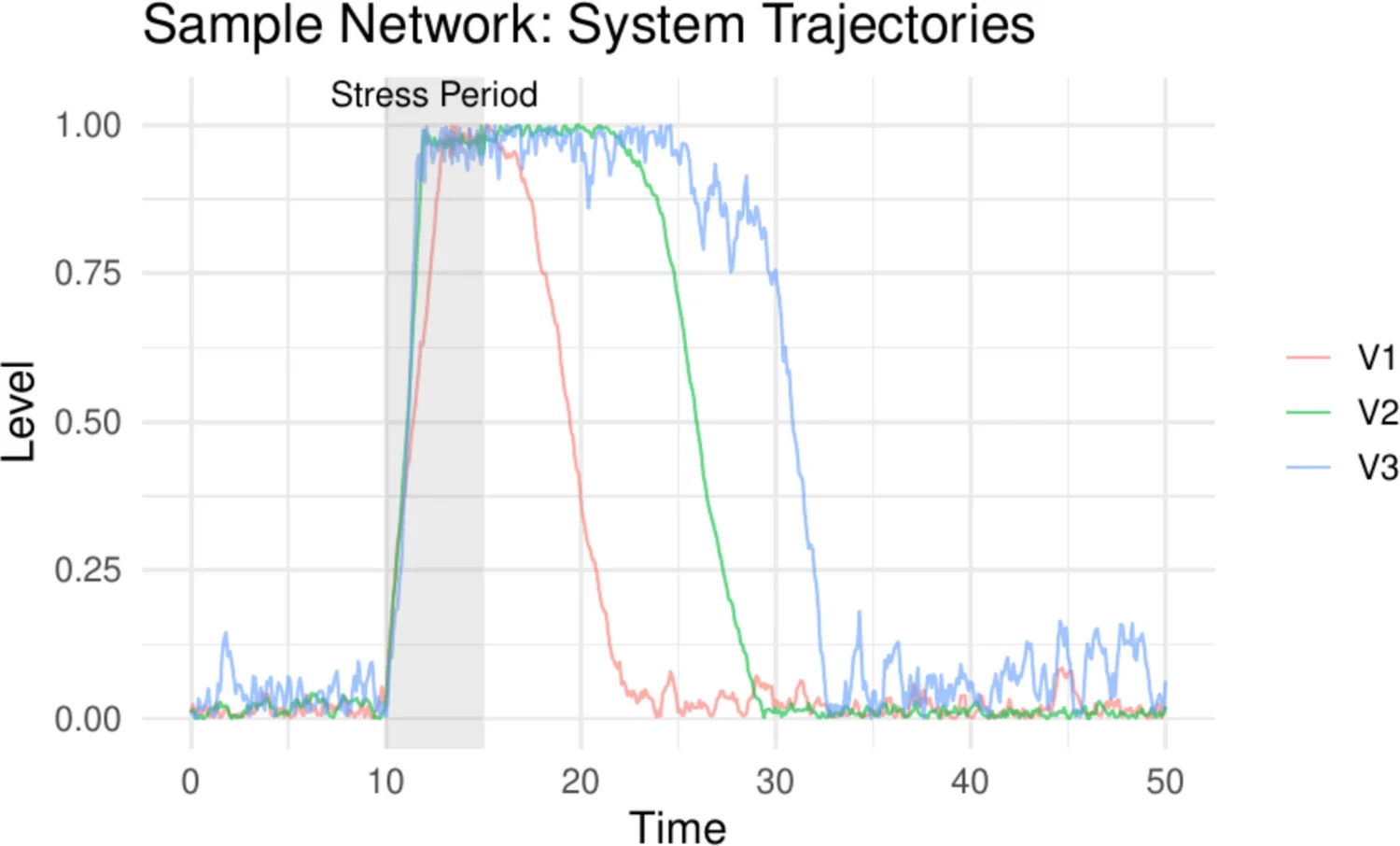
Example simulated trajectories from a single directed network under a temporary exogenous stress perturbation. The *shaded region* indicates the stress window (${\boldsymbol{t}}\in \left[10,15\right]$), during which each node receives an additive input of 0.05 per time step (see stress_event)

The output res is a time-by-node matrix containing the simulated system state over time. This output can be visualized using plot_dynamics(), as shown below; the resulting plot is displayed in Fig. 3.

**Figure:**
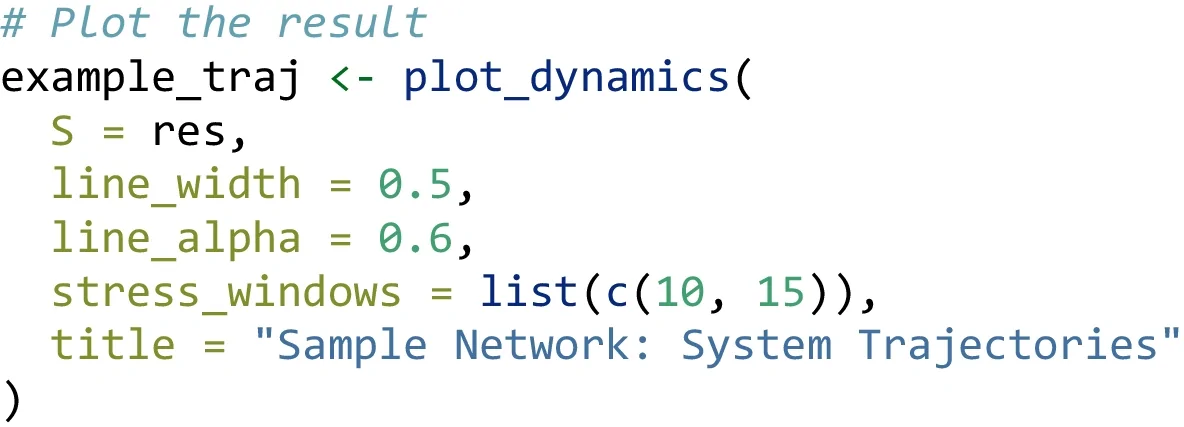

## Comparing dynamics: Loop-free vs. loop-rich networks

To illustrate how simulation can be used to explore structure–function relationships and to briefly review the full workflow, we compare two networks with differing feedback architectures, drawn from the three-node example. Specifically, we simulate system dynamics on one network with no feedback loops and another with the maximum number of loops. This example demonstrates how causal structure alone, holding all other factors constant, can meaningfully shape system behavior over time. This mirrors common clinical hypotheses about self-reinforcing symptom maintenance: feedback-rich architectures can sustain activation after a transient stressor.

To identify networks that differ in feedback structure, we begin by scanning the full set of enumerated configurations from the triangle skeleton. We use the detect_feedback_loops() function from the causalnet package, which takes a directed adjacency matrix as input and returns a list of unique feedback loops. We apply this function to all networks and extract two representative examples, one with no feedback loops and the other with the maximum number:

**Figure:**
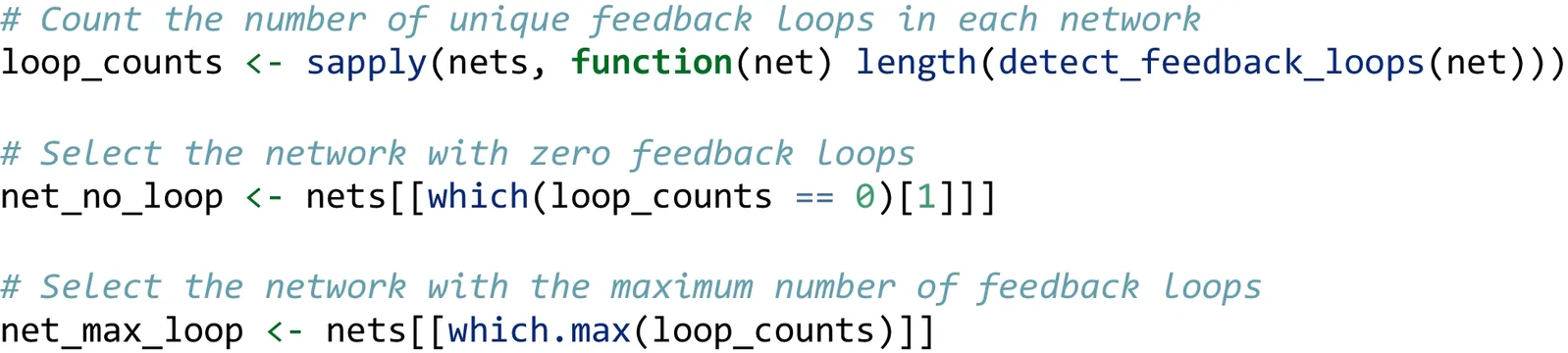

**Fig. 4:**
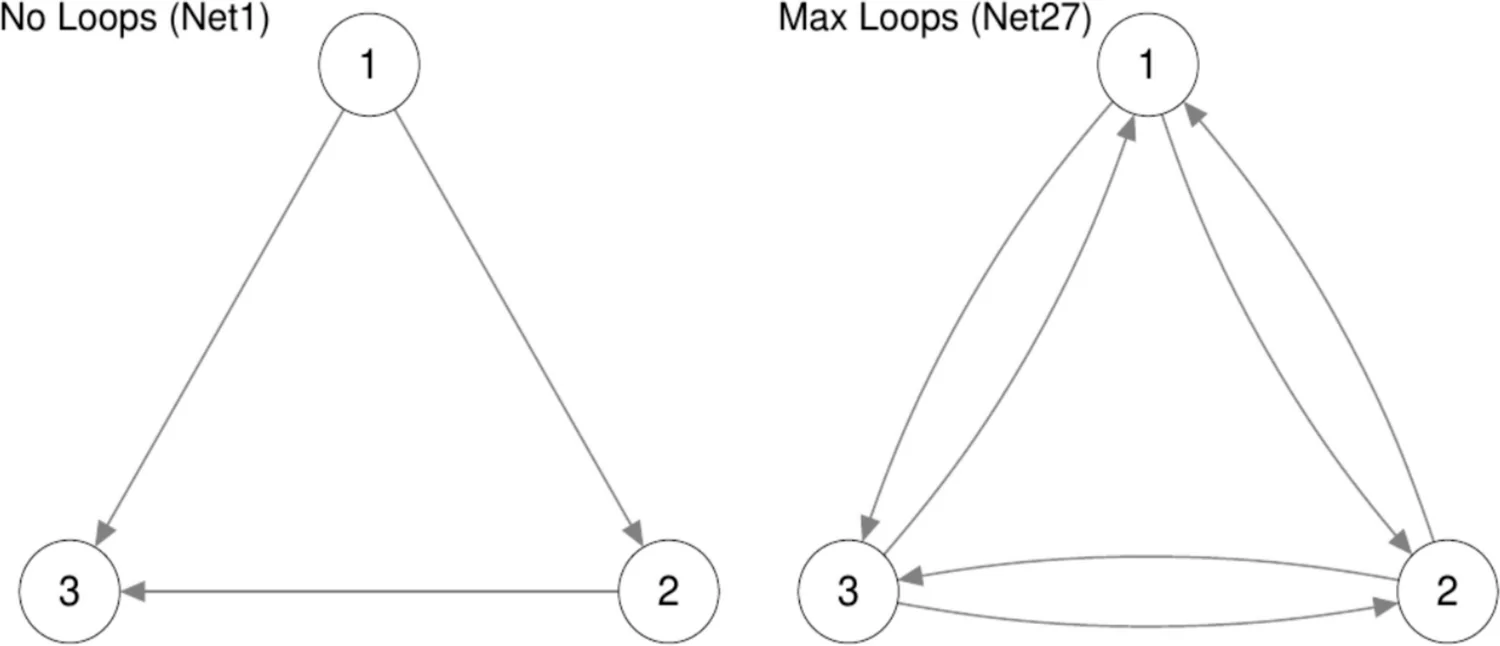
Representative networks: one with no feedback loops (*left*), and one with maximal feedback loops (*right*). Both networks are consistent with the same undirected skeleton

The two network structures are shown in Fig. 4. Although both networks share the same undirected skeleton and node identities, they differ in how edges are oriented: one is fully acyclic, while the other contains the maximum possible number of feedback loops. To isolate the influence of structure on dynamic behavior, we apply an identical set of parameters to both networks, sampled using get_sample_parameters():

**Figure:**
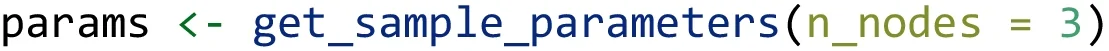

Next, we define a brief external perturbation that uniformly increases all node values by 0.05 between timepoints 10 and 15:

**Figure:**
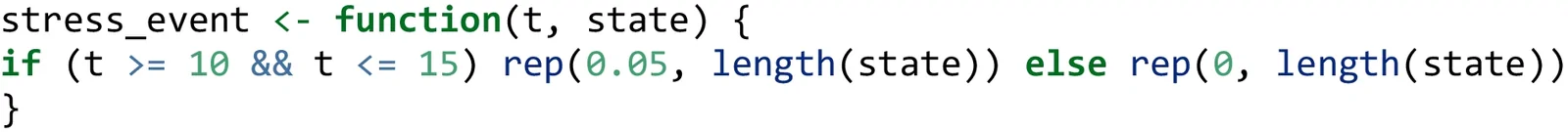

We then simulate system dynamics for both networks using the simulate_dynamics() function:

**Figure:**
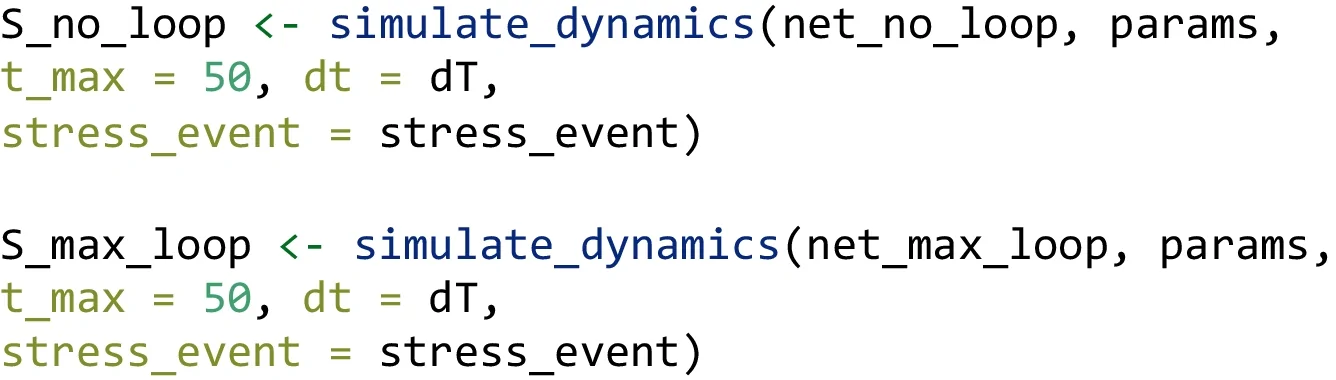

We can visualize the resulting dynamics using the plot_dynamics() function:

**Figure:**
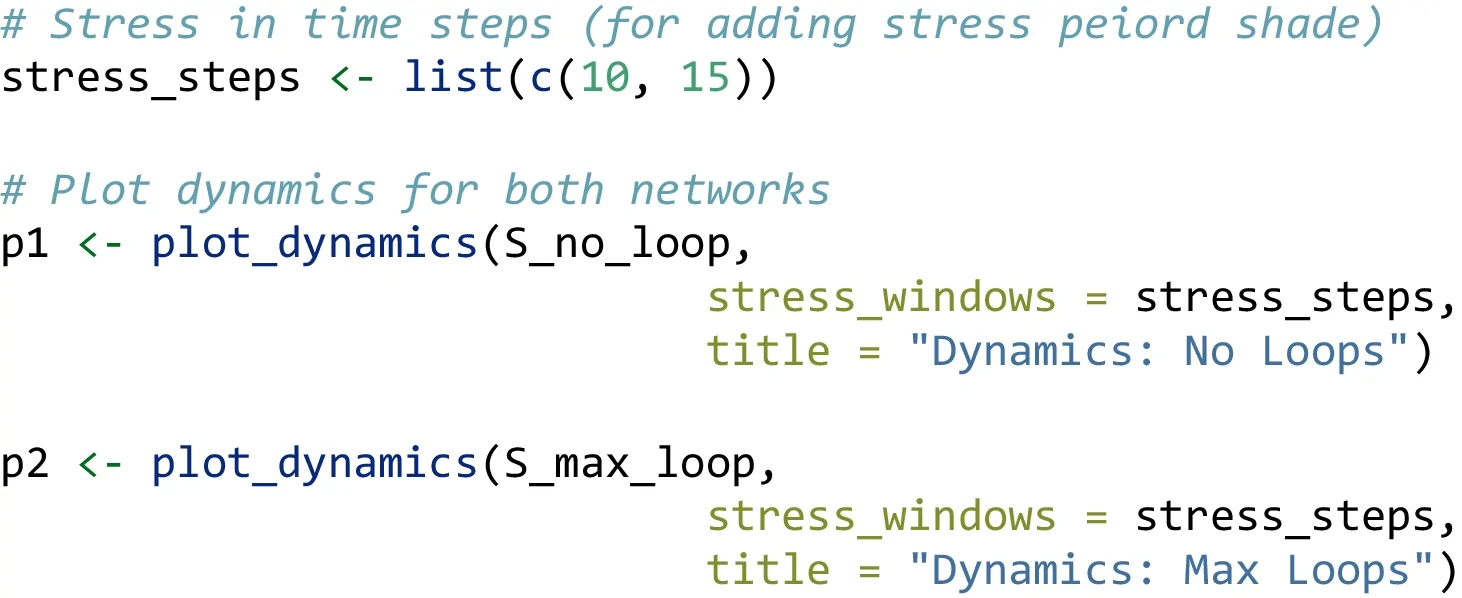

**Fig. 5:**
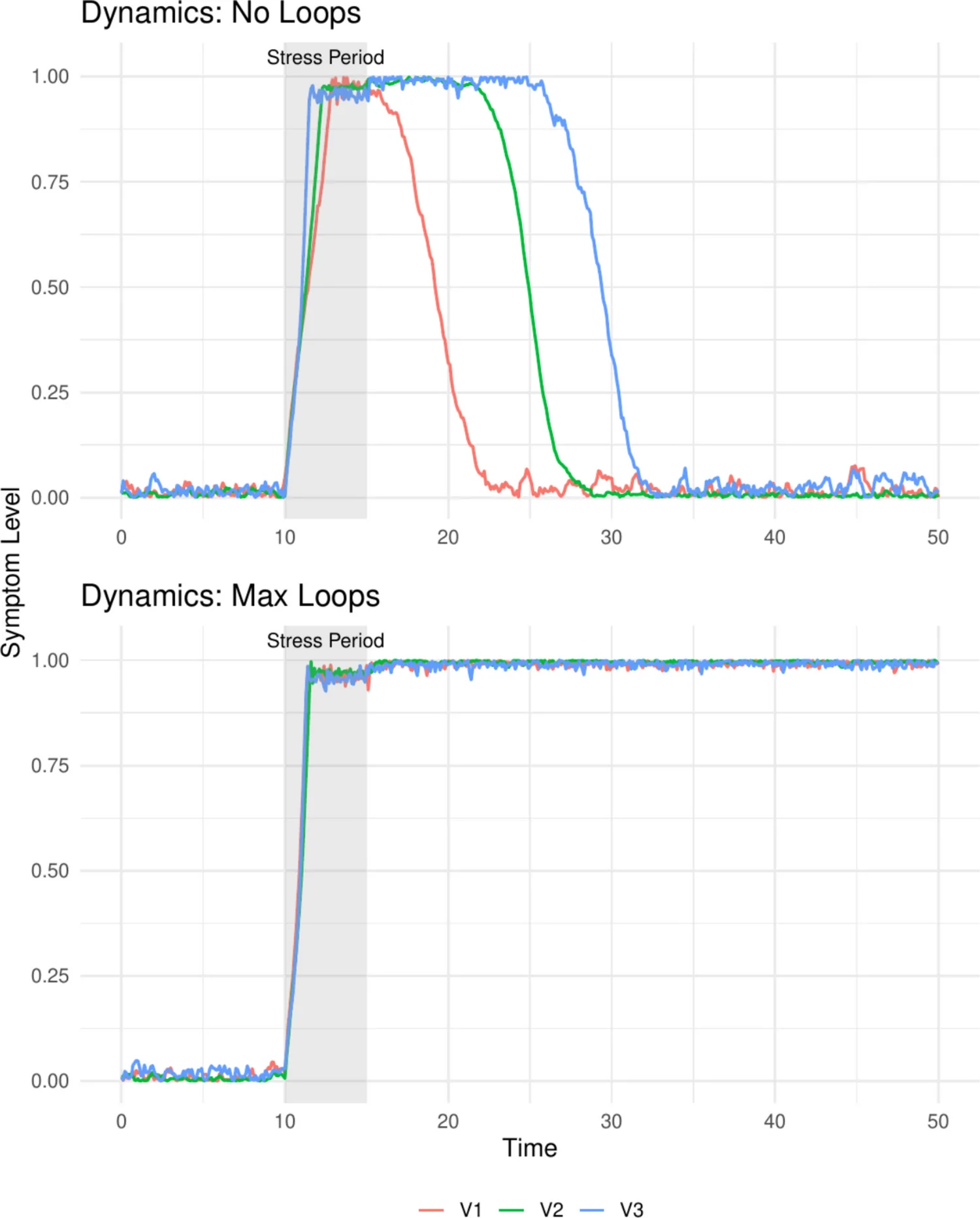
Simulated symptom dynamics for networks with no feedback loops (*top*) and maximal loops (*bottom*). The loop-rich network sustains higher activation levels and does not decay, illustrating how feedback structure can affect system persistence

The resulting dynamics for both network structures are shown in Fig. 5. Despite identical parameter values and stress input, the two networks display different patterns of system behavior. The loop-free network returns to baseline relatively quickly after the stressor is removed, with symptom levels stabilizing at low values. In contrast, the loop-rich network shows prolonged activation, with elevated symptom levels persisting for a longer period and showing no recovery during that time frame.

## Applied example: Modeling symptom networks

To demonstrate the practical application of the causalnet package, we analyze a real-world clinical dataset on chronic depression (Schramm et al. 2017). The study included 254 patients who received psychotherapy in Germany. In each treatment session, patients were assessed on nine core symptoms of major depressive disorder (Schramm et al. 2017), coded as binary indicators (0 = absent, 1 = present) aligned with DSM-5 diagnostic criteria (Schumacher et al. 2023). This applied example instantiates the same workflow introduced above—skeleton → constraints → admissible directed ensemble → architecture metrics → simulation-based comparison—using symptom data.

Our aim is to estimate a cross-sectional symptom network to serve as the structural skeleton, impose directional constraints, and then use causalnet to explore the space of consistent directed networks. Finally, we simulate and compare the dynamics of the system in different network configurations, parameter sets, and model types to illustrate how structural and dynamic properties jointly shape psychological processes.

## Step 1: Estimate a skeleton network

**Fig. 6:**
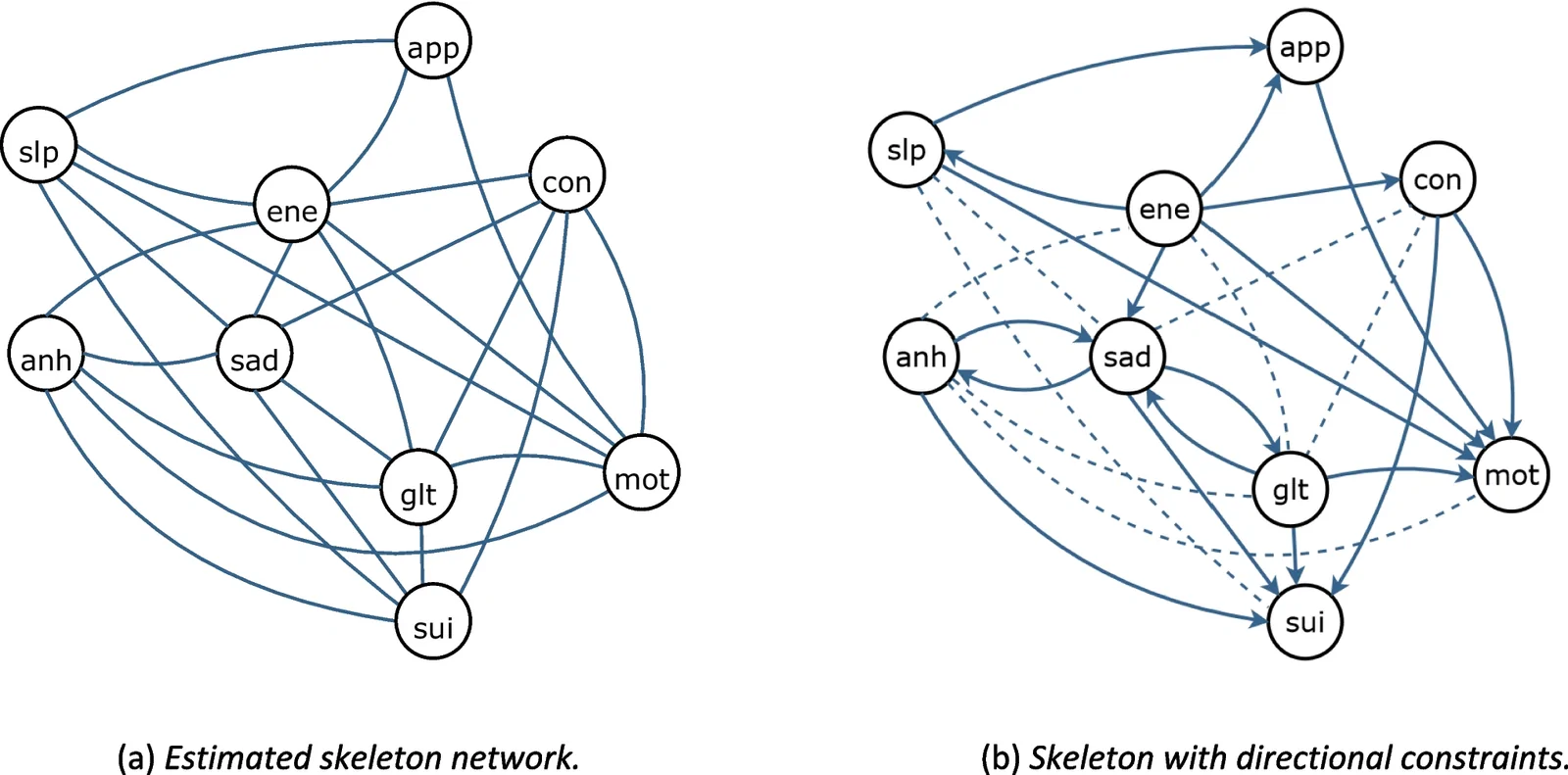
Estimated partial correlation network (a) and partially directed skeleton with constraints (b). *Solid arrows* represent imposed directional constraints; *dashed edges* indicate unconstrained links that are free to vary during network enumeration

We begin by estimating a cross-sectional symptom network at baseline, using nine binary items corresponding to the DSM-5 criteria for major depressive disorder. Given the dichotomous nature of the data (0 = symptom absent, 1 = present), we use the IsingFit method from the bootnet package, which models conditional dependencies between binary variables via a pairwise Markov random field. To focus on stronger associations and reduce model complexity, we retain only edges with absolute weights greater than 0.3. This filtered adjacency matrix defines the undirected skeleton used in subsequent steps to generate, constrain, and simulate directed networks. The final skeleton is visualized in Fig. 6a.

Here, the IsingFit model is used only to estimate an undirected association skeleton—that is, which symptom pairs show conditional dependence in the cross-sectional data. This step is used to define a candidate edge set and does not imply that the subsequent simulations follow Ising dynamics or that the Ising model is taken as the data-generating process. The simulation model used in later steps is a separate, user-chosen modeling assumption: it evolves node state variables on a bounded continuous scale $\left[0, 1\right]$ and is used as a flexible framework for comparing how alternative admissible directed structures produce different dynamic regimes under explicit assumptions. When researchers require simulations that align with binary symptom measurement, causalnet supports user-defined model_fn specifications, including discrete-time logistic/Bernoulli updating with states in 0,1 (see Appendix, Example A).

**Figure:**
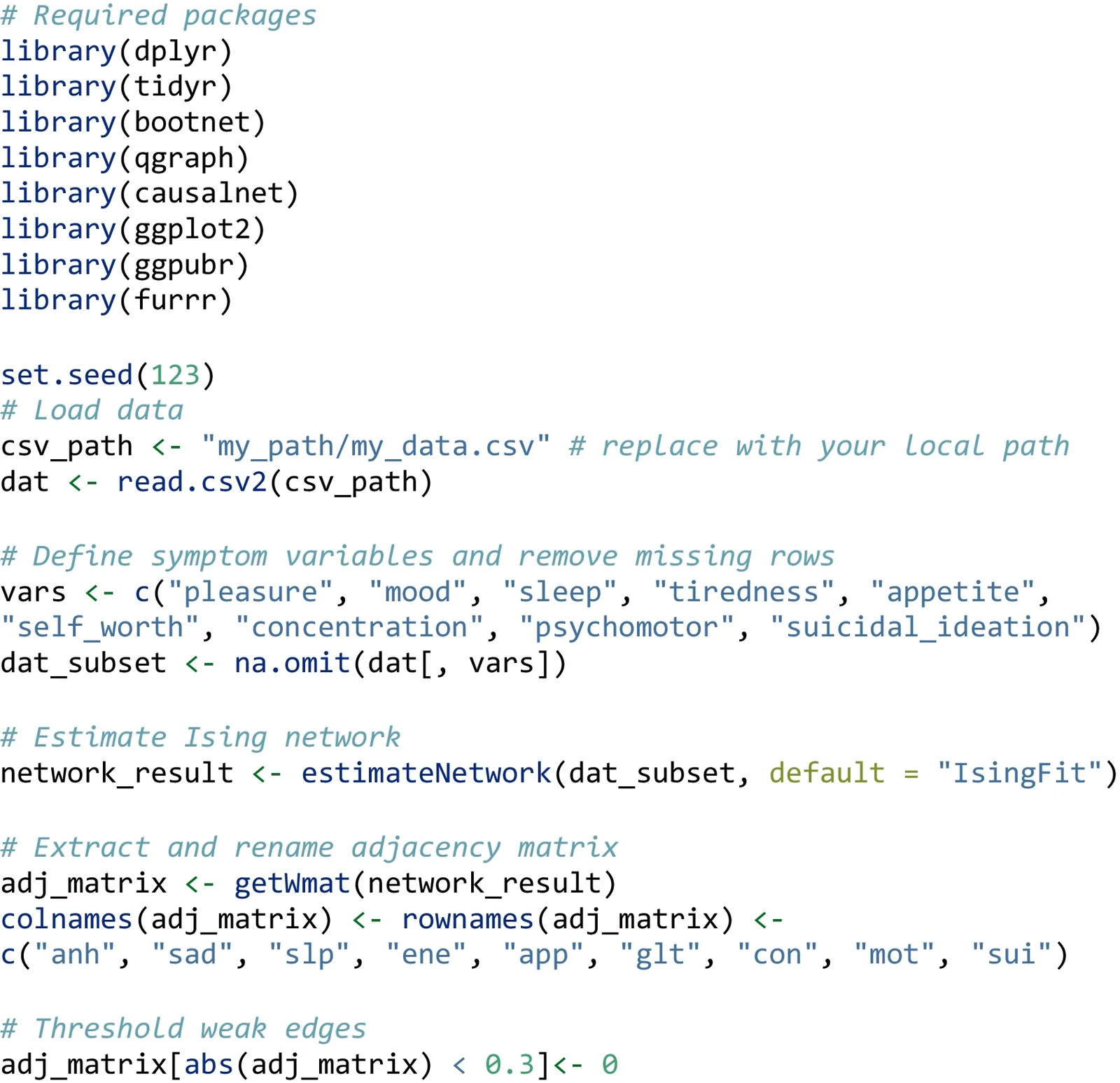

## Step 2: Add directional constraints

To illustrate how researchers might incorporate theoretical or empirical knowledge, we add a set of directional constraints based on prior work. Park et al. (2026b) applied a time-series causal discovery method to the same dataset and identified several robust directional effects. We also incorporate theory-motivated assumptions based on clinical intuition, for example, low self-worth (glt) contributing to suicidal ideation (sui), rather than the other way around.

These directional assumptions are encoded in a fixed_edge matrix, which is used to restrict the space of admissible directed networks during the enumeration. The matrix follows a simple convention: fixed_edge[i, j] = 1 indicates a required directed edge from node i to node j (i.e., i→j), fixed_edge[i, j] = 2 and fixed_edge[j, i] = 2 indicate a required bidirectional edge between nodes i and j (i.e., dyadic feedback loop), fixed_edge[i, j] = NA (the default) means no constraint, so the direction (if the edge exists) is left unconstrained.

To implement this, we first define a list of directional assumptions informed by prior modeling results and clinical reasoning. For example, we enforce bidirectional edges between sad and anh, and between sad and glt, to reflect mutual influence, that is, dyadic loops between these pairs of symptoms. The following R code demonstrates how to construct and populate the constraint matrix based on these assumptions:

**Figure:**
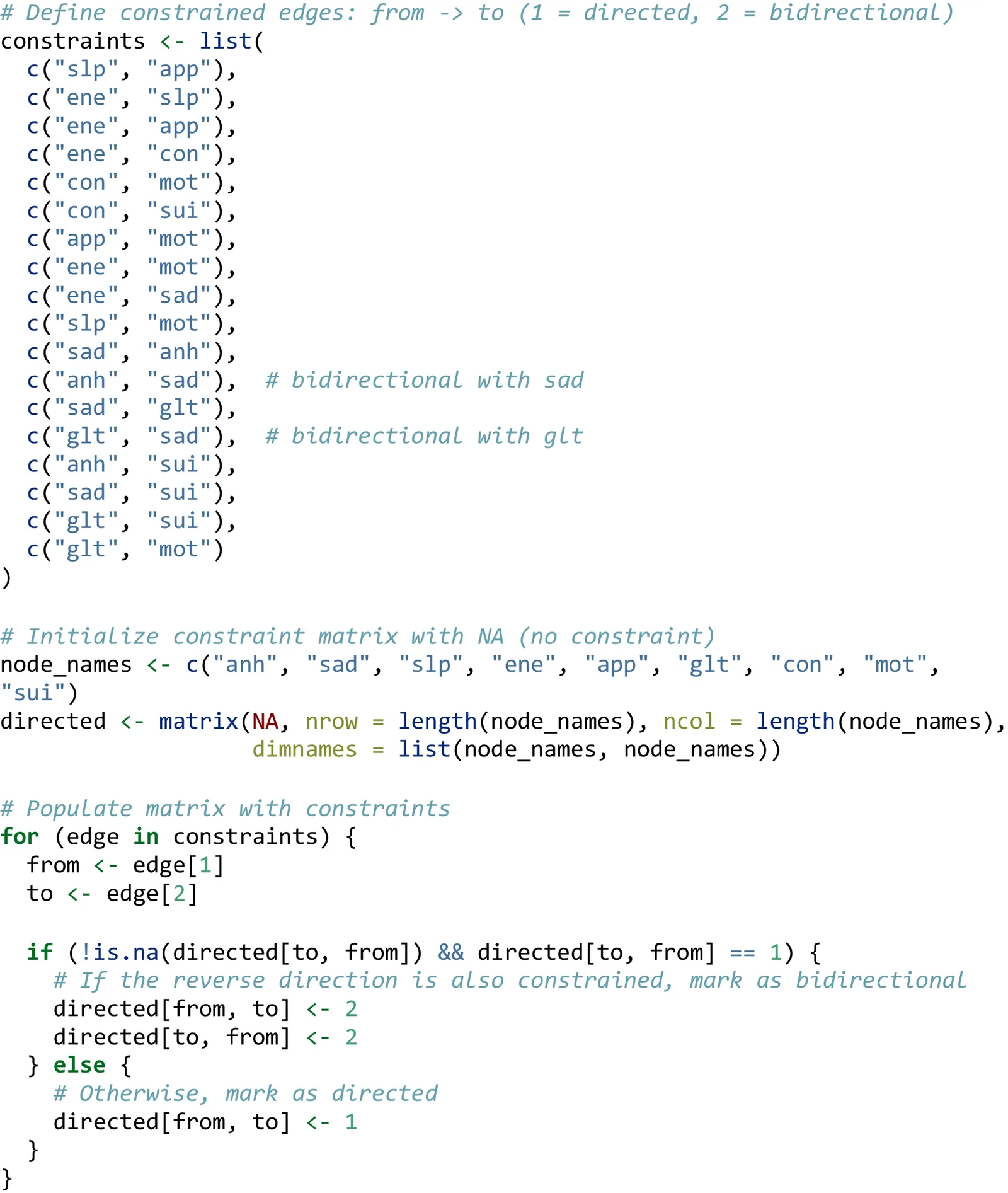

This constraint matrix is passed to the generate_directed_networks() function, restricting the search space to directed networks that satisfy both the undirected skeleton and the specified directional assumptions. Figure 6b illustrates the resulting hybrid structure: Constrained edges are shown as solid arrows, while unconstrained links remain undirected and appear as dashed lines.

## Step 3: Generate consistent directed networks

With the constrained skeleton in hand, we use the causalnet package to enumerate all directed networks consistent with the undirected structure and the imposed directional constraints. These candidate networks vary only in the direction of unconstrained edges.

The total number of possible networks depends on how many edges remain unconstrained. In our case, the skeleton and constraint matrix yield 6561 valid directed configurations, corresponding to all ${3}^{8}$ combinations of directions for the eight unconstrained edges. Each network is returned as a directed adjacency matrix, representing one admissible directed causal structure under the specified assumptions. Because full enumeration can be memory intensive, especially for larger networks, the generate_directed_networks() function includes a max_networks argument that restricts the number of networks returned. When this option is used, the function simply returns the first n networks generated in the internal enumeration order, without prioritization or random sampling. This feature is useful when working with large-scale systems or limited computational resources. Further customization options are described in the function documentation.

**Figure:**
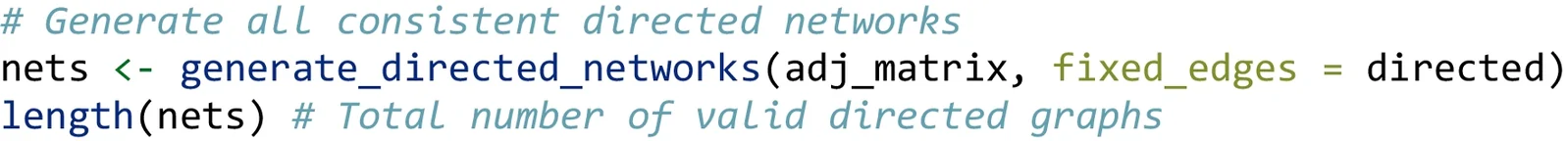

**Fig. 7:**
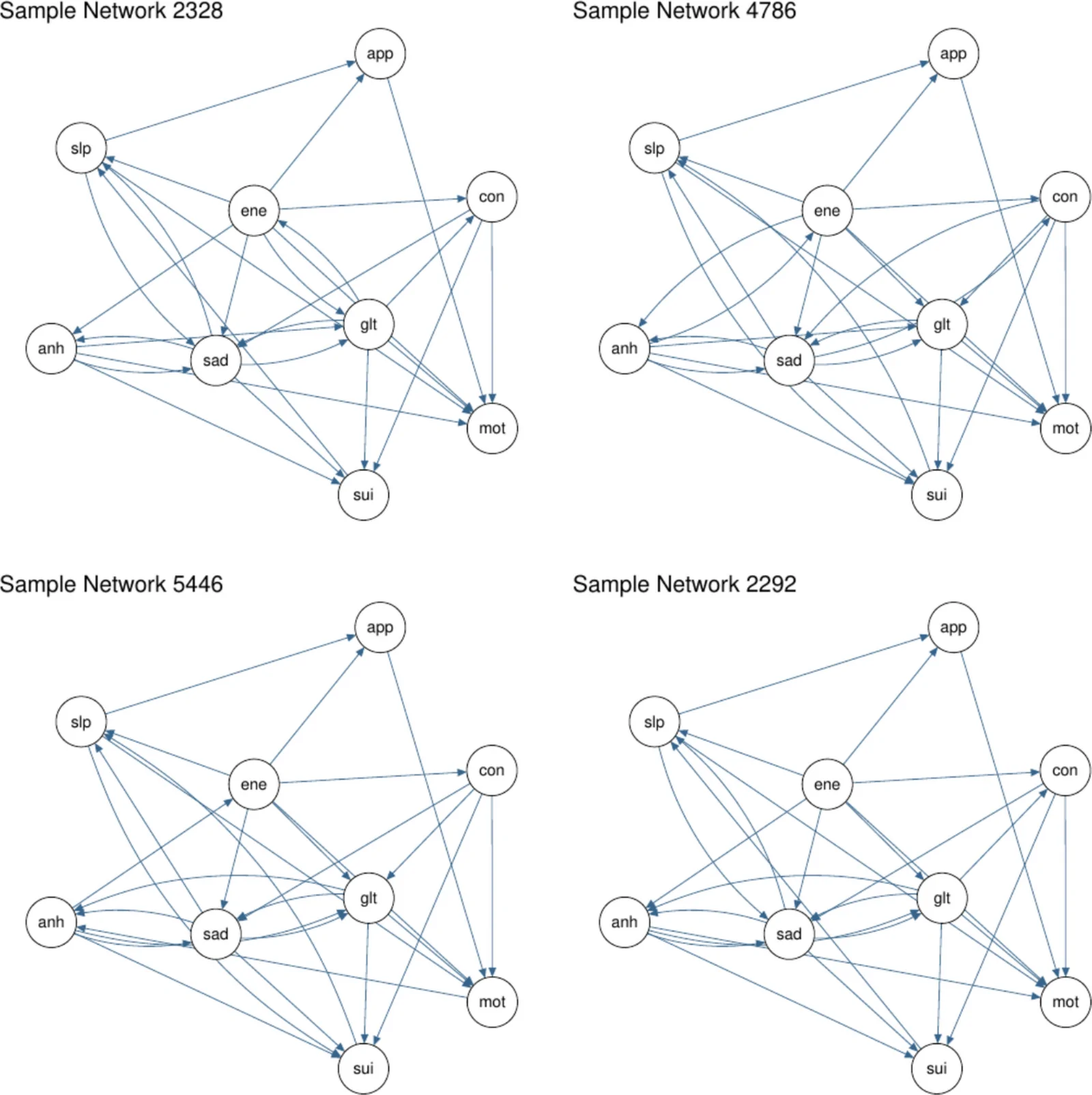
Example directed networks consistent with the constrained skeleton. Each panel shows one possible resolution of the ambiguous edges. These networks vary only in how unconstrained directions are assigned

These networks can be visualized directly using tools such as qgraph. For example, the code below randomly selects four networks from the generated list and plots them for inspection, as illustrated in Fig. 7.

**Figure:**
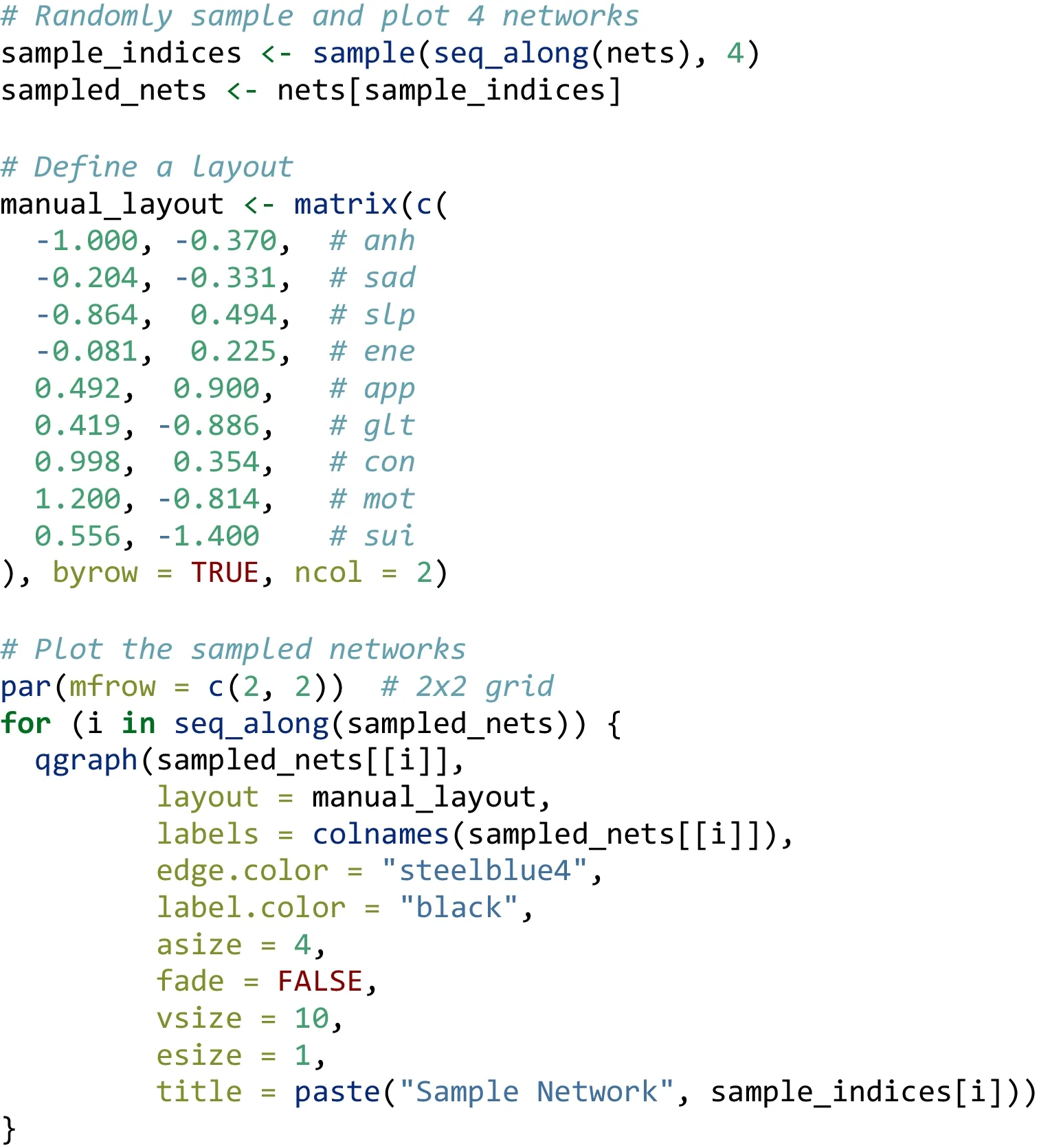

## Step 4: Analyze network architecture

After generating a set of directed networks consistent with the estimated skeleton and directional constraints, an optional next step is to explore how these networks differ in their structural organization. This analysis is not required for all use cases but may be informative if users are interested in architectural variation across networks. Even when edge density and directional constraints are fixed, the resulting topologies can vary widely in their connectivity patterns.

To quantify such differences, the function summarize_network_metrics() provides a concise summary of four key topological features described earlier. These metrics are useful for comparing networks at the structural level, but users are not limited to them; other network characteristics can be computed depending on the research question. The code below demonstrates how to generate and visualize these summaries:

**Figure:**
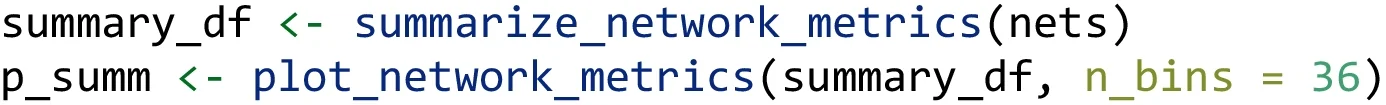

**Fig. 8:**
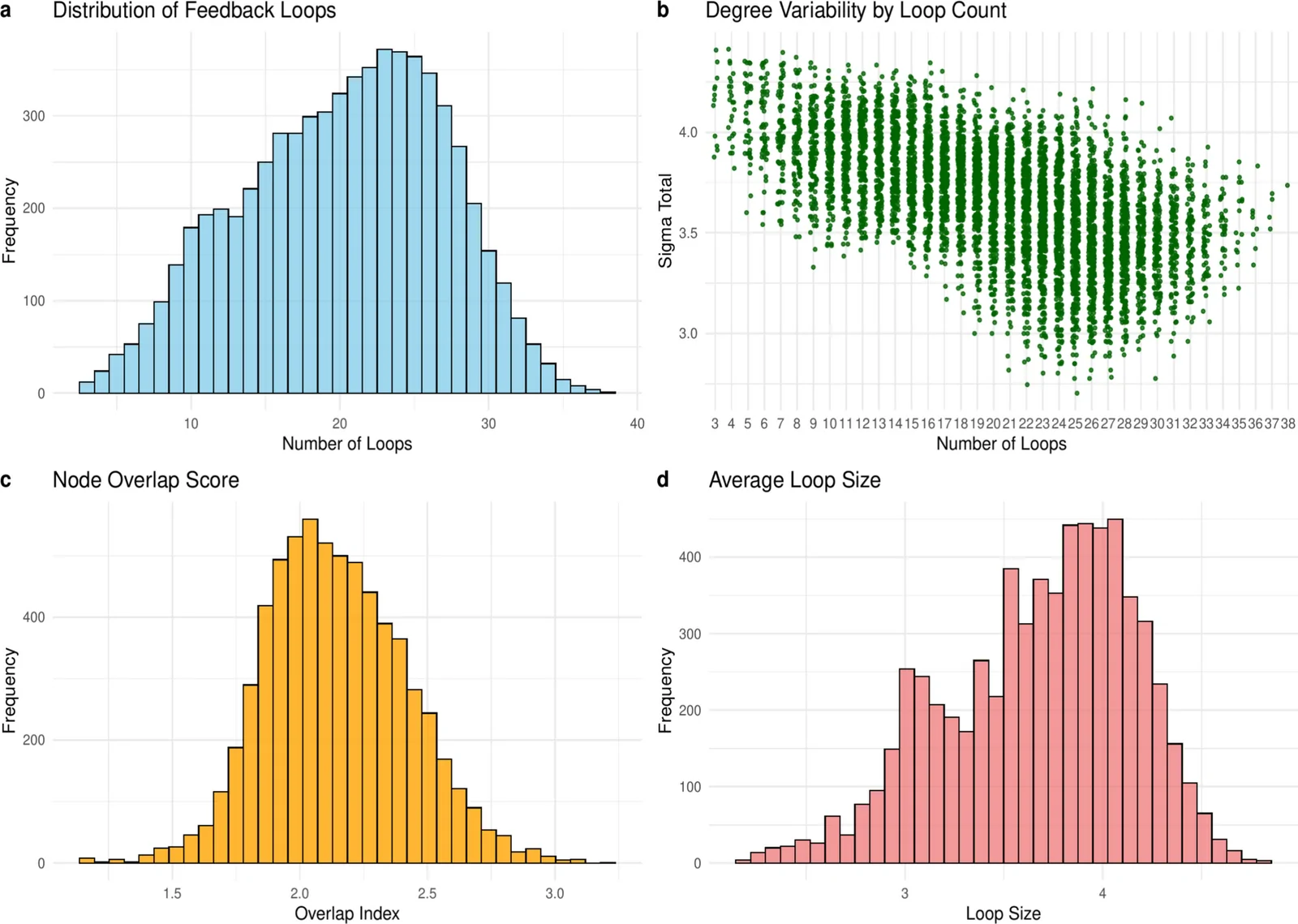
a–d Distribution of structural metrics across all directed networks consistent with the constrained skeleton. Although edge density and directional constraints are fixed, network architecture varies considerably

Figure 8 shows substantial variation in structural properties across the 6561 networks. Importantly, the four panels should be interpreted jointly as an “architecture profile” of the ensemble: Fig. 8a summarizes how much feedback is present (loop count), Fig. 8d summarizes the typical scale of feedback (average loop size), and Fig. 8b, c describes how this feedback is organized across nodes (degree heterogeneity and concentration of loop participation). In Fig. 8a, we see that the minimum number of feedback loops is three, due to bidirectional edges explicitly fixed in the skeleton (e.g., between sad and anh, and sad and glt). Loop counts extend to a maximum of 38, indicating that many additional feedback cycles arise automatically when unconstrained edges are oriented in particular ways, beyond the bidirectional edges explicitly fixed in the skeleton.

Interestingly, degree variability tends to show a modest negative relationship with loop count (see Fig. 8b). This may reflect a structural trade-off: networks with many feedback loops typically distribute edges more evenly across nodes, as forming cycles requires that many nodes both send and receive connections. Such configurations reduce the disparity in node degrees, resulting in lower variability. In contrast, networks with fewer loops often exhibit more chain-like or hierarchical topologies, where influence is funneled through a limited number of hub nodes—some of which dominate as primary sources or sinks—thereby inflating degree variance.

In Fig. 8c, the node overlap score, which measures how often nodes appear in multiple feedback loops, is roughly normally distributed. This suggests that feedback is neither highly localized to a few hubs nor uniformly spread, but occupies a middle ground across the ensemble.

Finally, Fig. 8d shows that most networks have an average loop size between 3 and 5 nodes. In the present n=9 network, this corresponds to loops involving roughly one-third to one-half of the system, indicating that feedback structures are typically local-to-intermediate in scale rather than spanning nearly all nodes.

Taken together, these patterns suggest that networks with more evenly distributed feedback—reflected in higher loop counts, lower degree variability, and moderate node overlap—may support more resilient or decentralized dynamics. Such structures avoid over-reliance on specific hubs and instead enable influence to circulate across multiple pathways. However, this is only one possible interpretation. The causalnet framework is intentionally flexible: Users can explore very different structural hypotheses, such as identifying networks with minimal feedback, isolating specific causal motifs, or comparing topologies that prioritize efficiency or modularity. Additional or customized metrics can be easily computed depending on the research question at hand.

## Step 5: Simulate symptom dynamics

The final stage of the workflow demonstrates how causalnet can be used for theory-driven model comparison using dynamic simulations. Specifically, we compare the dynamic signatures implied by directed network structures that are admissible given an estimated skeleton and a set of directional constraints (e.g., persistence versus recovery following a perturbation, or heterogeneity across symptoms) under explicit modeling assumptions. This step is intended to help applied researchers connect structural hypotheses (encoded as constraints) to predicted system behavior and evaluate which structural assumptions yield dynamics that are most consistent with domain expectations and, where available, empirical benchmarks.

In practice, this stage can be used as an iterative screening and model-checking workflow: (i) specify one or more target features of the phenomenon that the simulations should capture (qualitatively or approximately; e.g., marginal symptom levels, variability, autocorrelation, recovery time, or intervention response patterns); (ii) choose an update rule and parameterization appropriate to the measurement and time scale (default nonlinear model, linear alternative, or a user-supplied model_fn); and (iii) compare candidate structures (and, if relevant, parameter regimes) in terms of how well they reproduce these targets. The outcome of this comparison is itself informative: if no admissible structure matches key targets under reasonable parameterizations, this suggests that the current combination of skeleton/constraints and process-model assumptions is unlikely to be adequate and motivates revisiting constraints, changing the process model class, and/or refining how observed symptoms are linked to the simulated state variables. Conversely, if only a subset of admissible structures matches the targets, researchers can prioritize those candidates for interpretation and sensitivity analysis, and examine which structural assumptions they share and how robust the match is to parameter variation.

To illustrate this workflow, we simulate the dynamics of the symptoms in all 6561 networks consistent with the constrained skeleton. While we use the default setup, users are encouraged to adapt the simulation framework to their own research questions, for example, by modifying the model type, parameter ranges, or stress inputs.

## Simulation setup

**Fig. 9:**
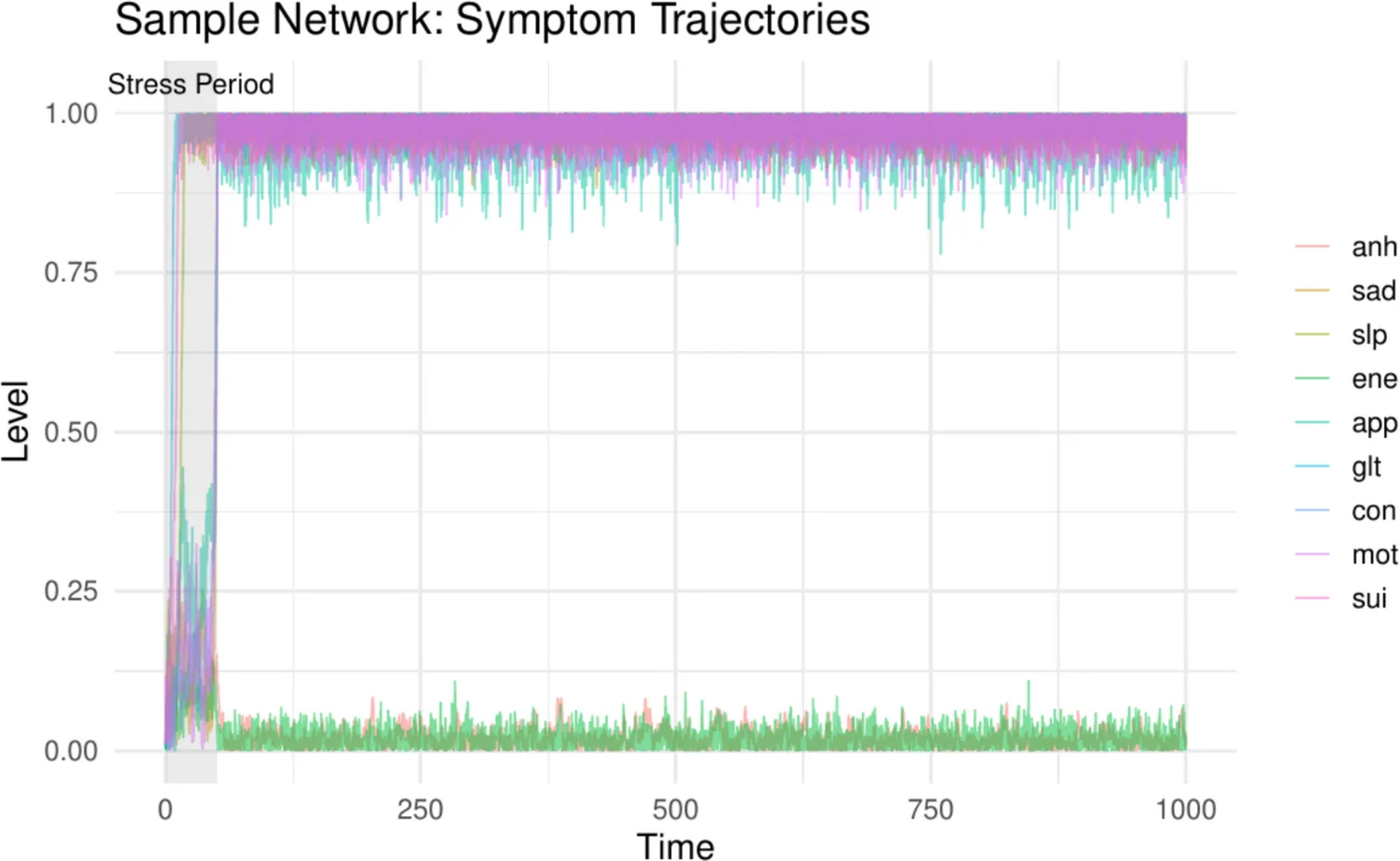
Simulated symptom trajectories for a single network under a brief stress perturbation. Each *line* represents a different symptom node. The *shaded region* marks the external stress period (*t* = 0–50). After the stress period, symptom dynamics unfold heterogeneously across nodes, with some symptoms showing persistence while others remain near the deactivated state

We first define a transient stress period between timepoints t=0 and t=50 (out of a total horizon ${t}_{\mathrm{m}\mathrm{a}\mathrm{x}}=1000$), during which all nodes receive a small external input. This perturbation is optional and fully customizable. Simulations use the default nonlinear model to generate bounded dynamics with intrinsic noise. The node-level parameters (β, ${\alpha}_{self}$, δ, σ) are drawn from the default ranges using get_sample_parameters(). As an illustration, we simulate the dynamics on an example network to examine how the system responds over time. Figure 9 displays the resulting trajectories. Following the stressor, the dynamics of the symptoms diverge between the nodes: some nodes return to the baseline, while others remain persistently elevated. The code below shows how to define the stress event, sample parameters, run the simulation, and visualize the trajectories:

**Figure:**
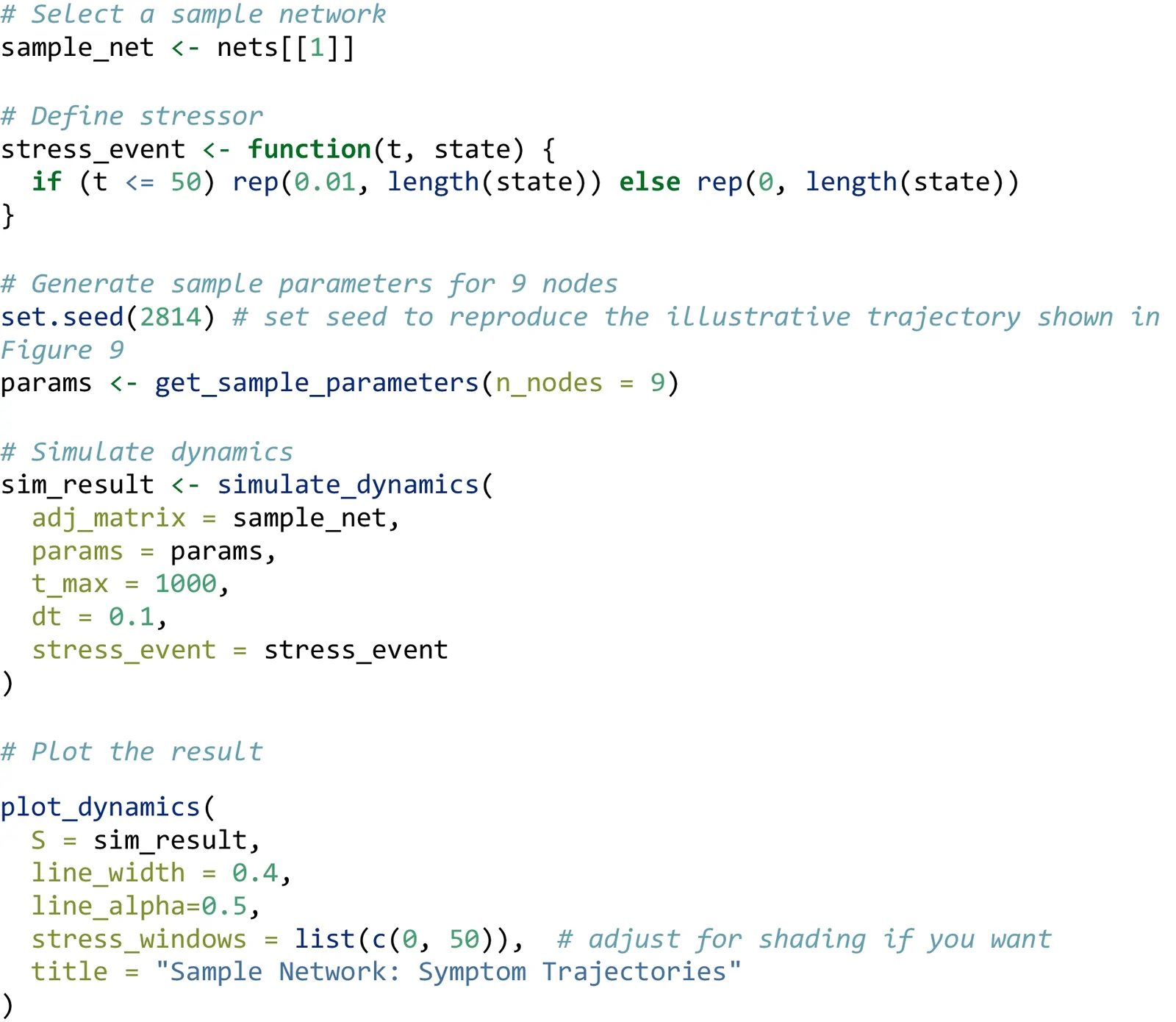

To systematically explore the ensemble, we simulate each of the 6561 networks 30 times, drawing a fresh set of parameters for each run. For each simulation, we extract two summary outcomes at the final time point: (1) the *mean symptom sum*, representing the total activation across all nodes, and (2) the *symptom level variability*, measured as the standard deviation of node activations. The following code demonstrates how this simulation pipeline is implemented, including parallelization for efficient computation. Because the full ensemble simulation can be computationally intensive, we also provide a saved.rds file containing the simulation results in the GitHub repository (causalnet/paper/results/applied_example_results.rds). Readers can load this file and follow the downstream analyses.

**Figure:**
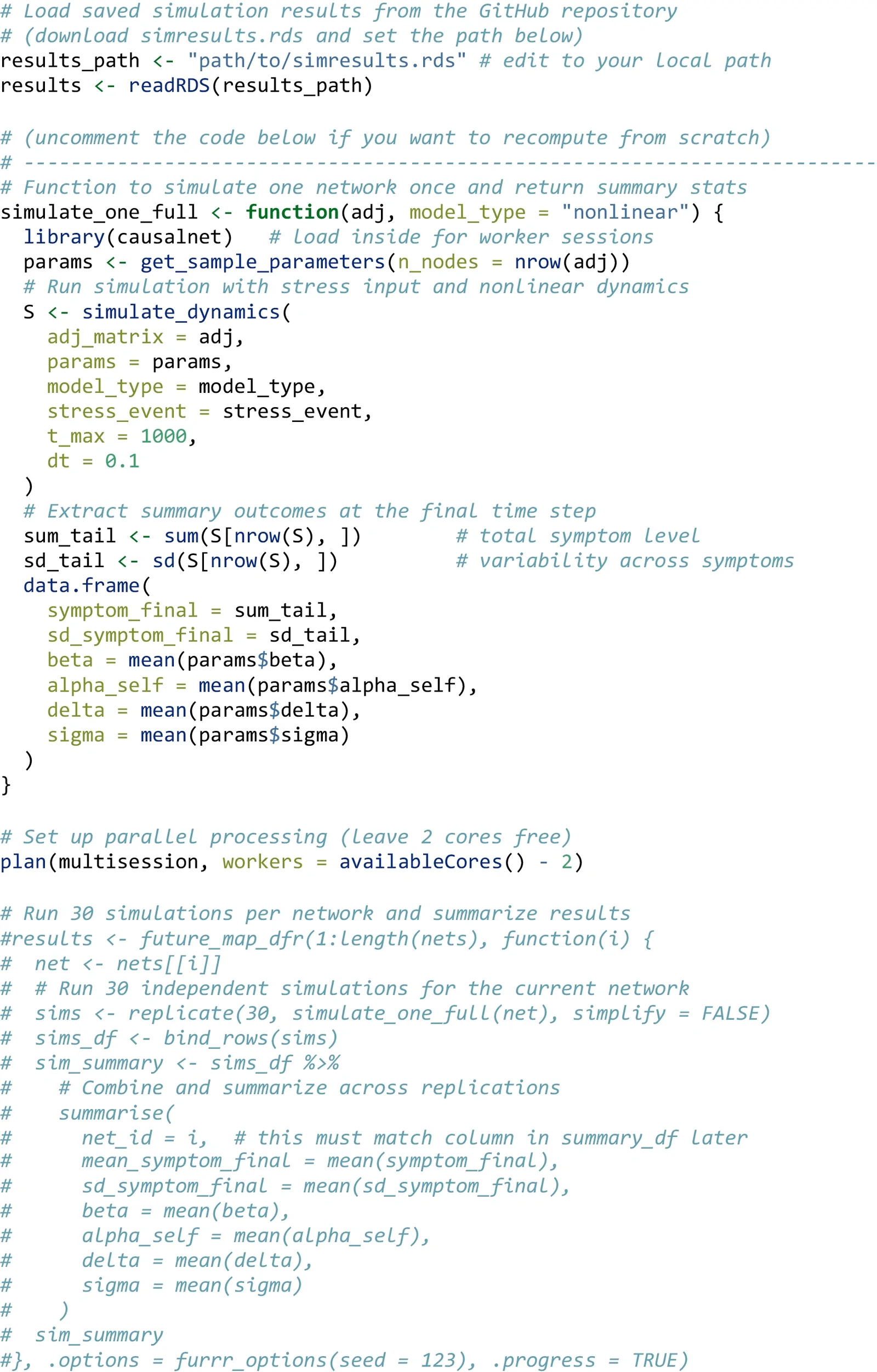

## Exploring the distribution of outcomes

**Fig. 10:**
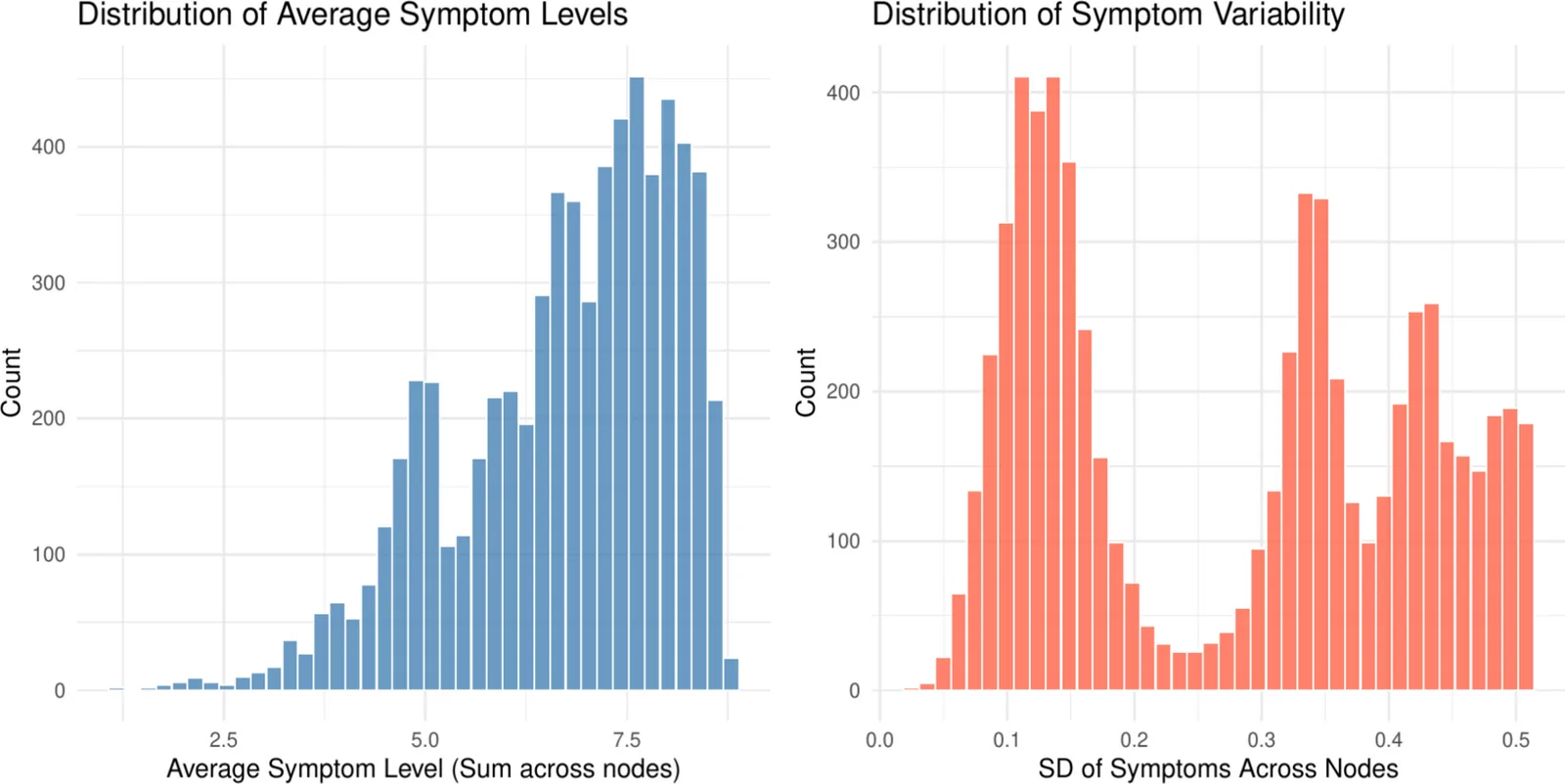
Distribution of average symptom levels (*left*) and across-node variability (*right*) at the final time point. Most networks sustain moderate-to-high activation levels, while variability across nodes shows a bimodal distribution.

Figure 10 summarizes two key dynamic outcomes measured across the full ensemble of simulated networks: (a) total system activation (i.e., the sum of symptom levels across nodes) and (b) symptom variability (i.e., the standard deviation across nodes) at the final time point. Most networks sustain moderately high activation levels after the stressor (sums between approximately 5 and 8), with a long tail toward lower values. In contrast, the distribution of across-node variability is distinctly bimodal, indicating that some networks produce relatively uniform symptom activation, while others yield heterogeneous profiles with marked differentiation across nodes. The code used to generate Fig. 10 is provided below.

**Figure:**
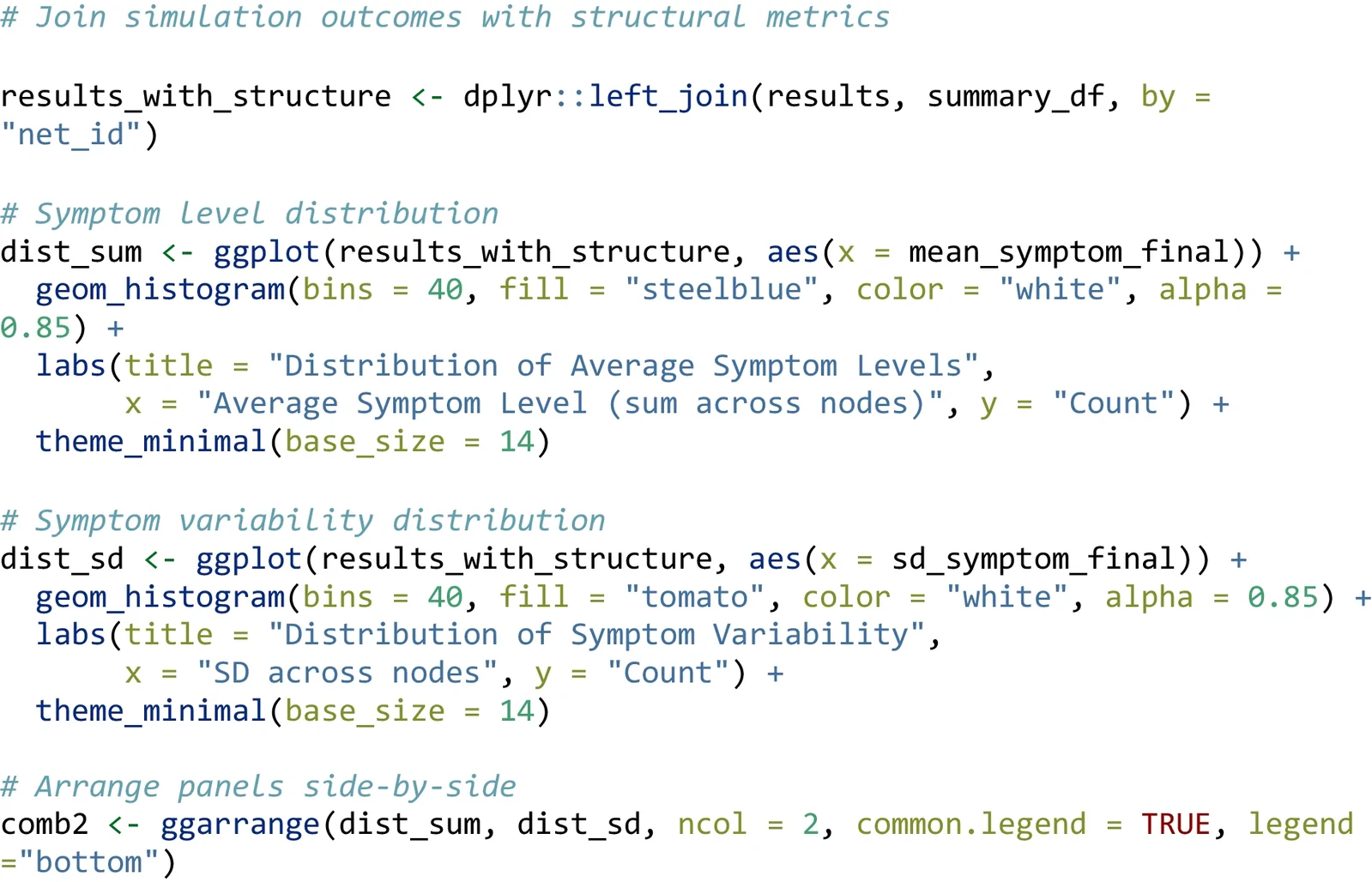

## Relating structure to dynamics

**Fig. 11:**
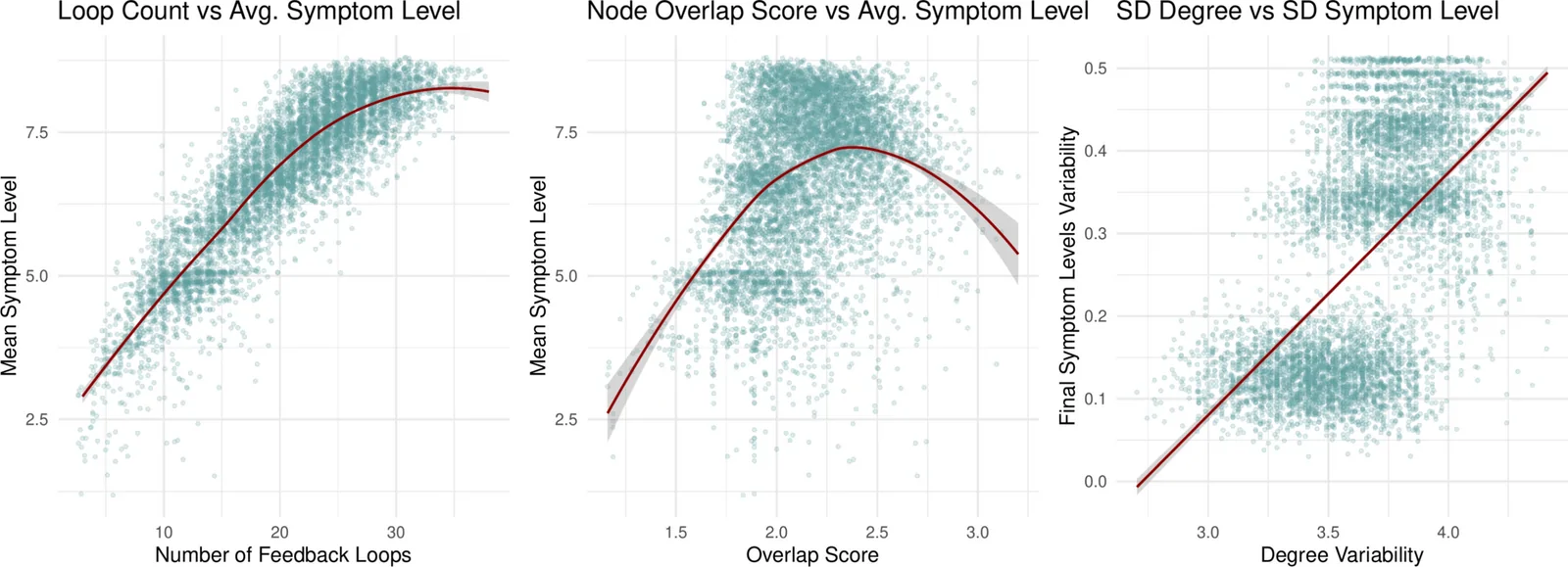
Relationships between structural features and dynamic outcomes. *Left*: feedback loop count vs. mean symptom sum; *Middle*: node overlap score vs. mean symptom sum; *Right*: degree variability vs. symptom variability. *Smooth curves* are LOESS fits. Networks with more feedback loops tend to sustain higher activation. The association between node overlap score and symptom level is non-monotonic but difficult to interpret in isolation, as it may reflect correlations with other structural features (e.g., loop count). Degree variability is positively associated with heterogeneity in node activation

To link dynamic outcomes back to the network architecture, users can relate dynamic summary measures to the structural features computed in Step 4. Figure 11 provides an initial impression of these relationships. Networks with more feedback loops tend to sustain higher average symptom levels. Degree variability is positively associated with symptom heterogeneity, suggesting that networks with greater node-to-node connectivity variability yield more differentiated activation patterns. The relationship between the node overlap score and symptom severity is less straightforward. Although networks with moderate overlap tend to show the highest average activation, this pattern is likely influenced by correlations with other features, such as loop count, and should be interpreted with caution.

These exploratory analyses can be extended in multiple directions depending on the research question. For example, users may fit linear or generalized additive models (GAMs), perform unsupervised clustering methods (e.g., k-means), or examine how specific structural motifs relate to dynamic signatures. The causalnet workflow is intentionally flexible and does not prescribe a specific modeling strategy. Additional structural or dynamic features can be calculated and tested based on the theoretical focus of the user and the behavioral patterns of interest. The following subsections illustrate two such analyses for demonstration purposes. The code used to generate Fig. 11 is provided below.

**Figure:**
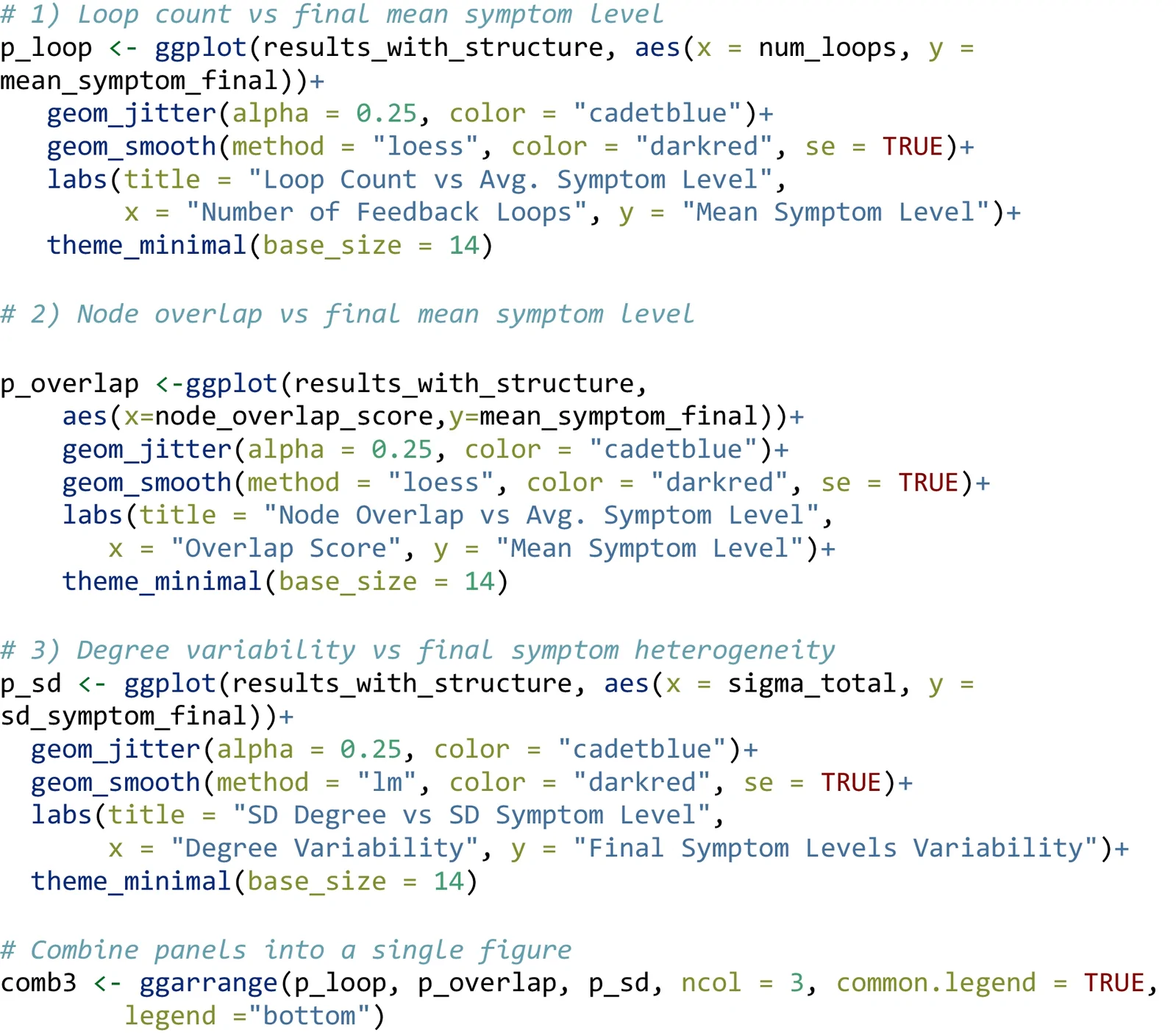

## Joint effects of structure on symptom patterns

**Fig. 12:**
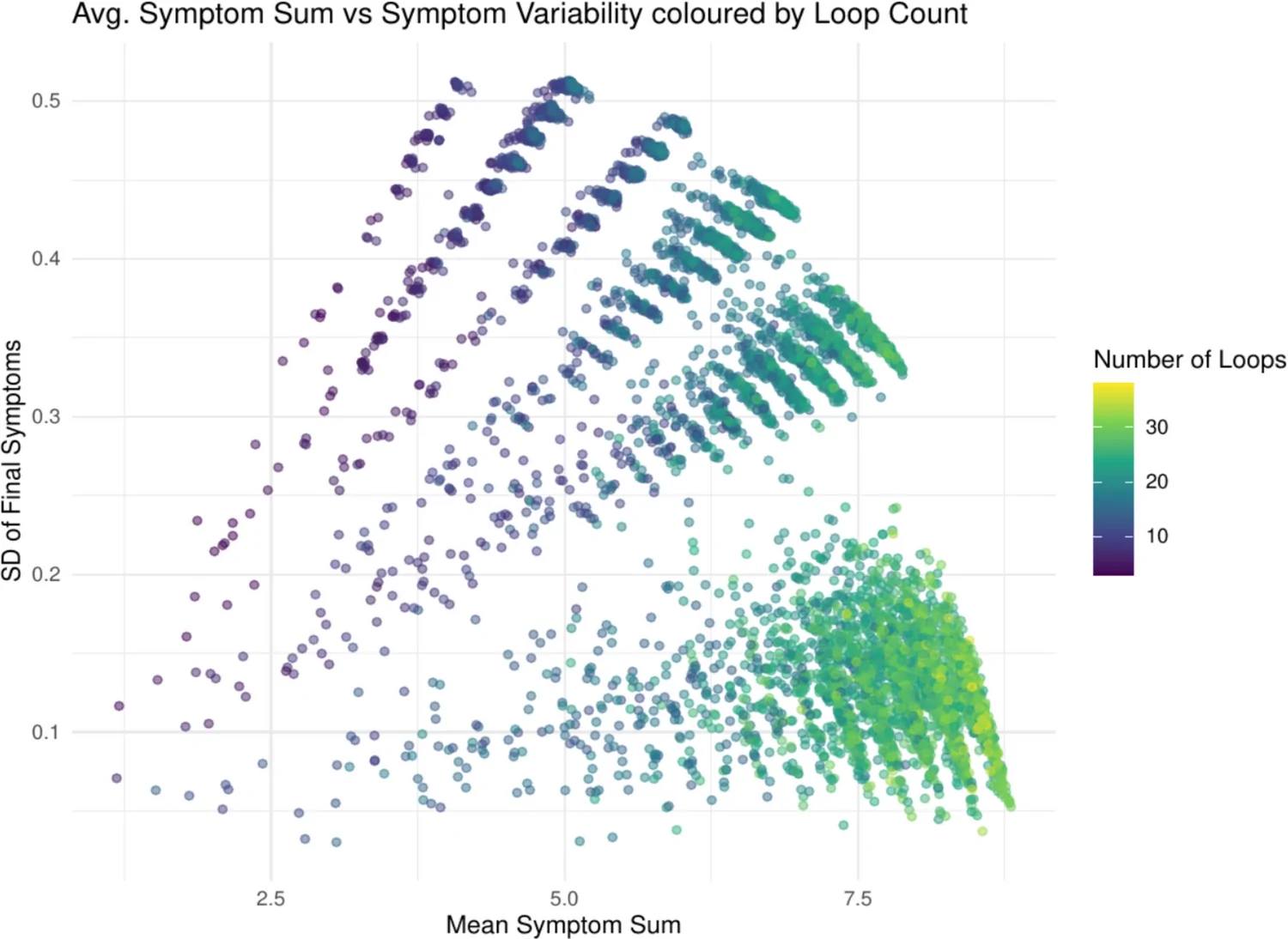
Networks plotted by mean symptom sum and symptom variability (SD), colored by feedback loop count. Higher loop count is associated with higher average symptom level and lower across-node variability. The pattern reveals a gradient of behavioral regimes tied to structural complexity

Beyond examining structure–outcome relationships one at a time, it can be informative to visualize how structural features jointly shape multiple aspects of system behavior. Figure 12 maps each network into a two-dimensional outcome space defined by the average level of symptoms (*x*-axis) and the variability of symptoms between nodes (*y*-axis), with points colored by the number of feedback loops. This visualization reveals an interesting pattern. Networks with more feedback loops (shaded from green to yellow) tend to cluster in the upper-left region, characterized by high overall symptom levels, but low variability. This suggests a regime of globally elevated, yet relatively homogeneous, activation. In contrast, networks with fewer loops (shown in purple) span a broader range, including low-activation states with greater heterogeneity across nodes. These results highlight how increasing feedback complexity shifts the system toward a more uniformly activated state, reinforcing the role of feedback architecture in shaping collective system dynamics. The code used to generate Fig. 12 is provided below.

**Figure:**
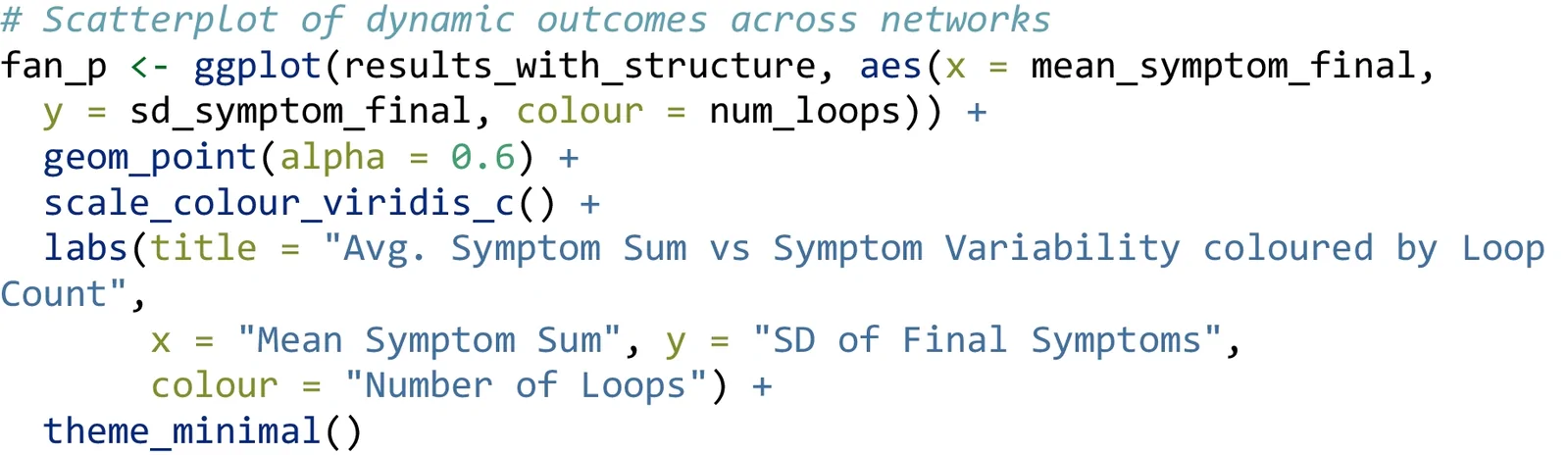

## Modeling nonlinear effects with GAM

Users may be interested in performing regression analyzes to relate structural features and model parameters to dynamic outcomes such as mean symptom levels. As the scatterplots above suggest, many of these relationships appear to be nonlinear. In such cases, one can fit a Generalized Additive Model (GAM), which models each predictor via a smooth function of its value.^2^ Below is an example of how users can apply a GAM to examine how structural and parametric features relate to mean symptom levels. Each predictor enters the model via a smooth term using the s() function.^3^

**Figure:**
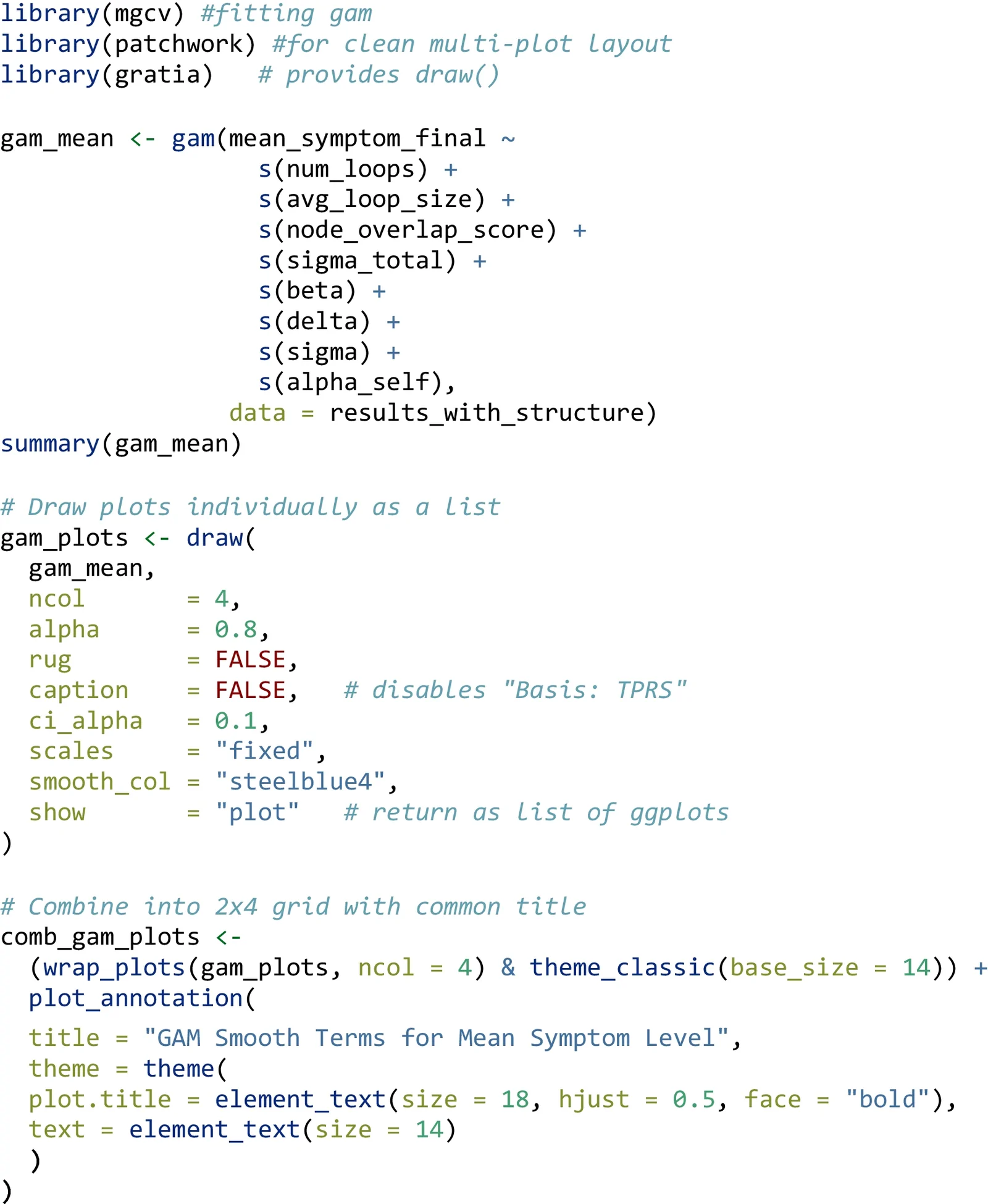

**Fig. 13:**
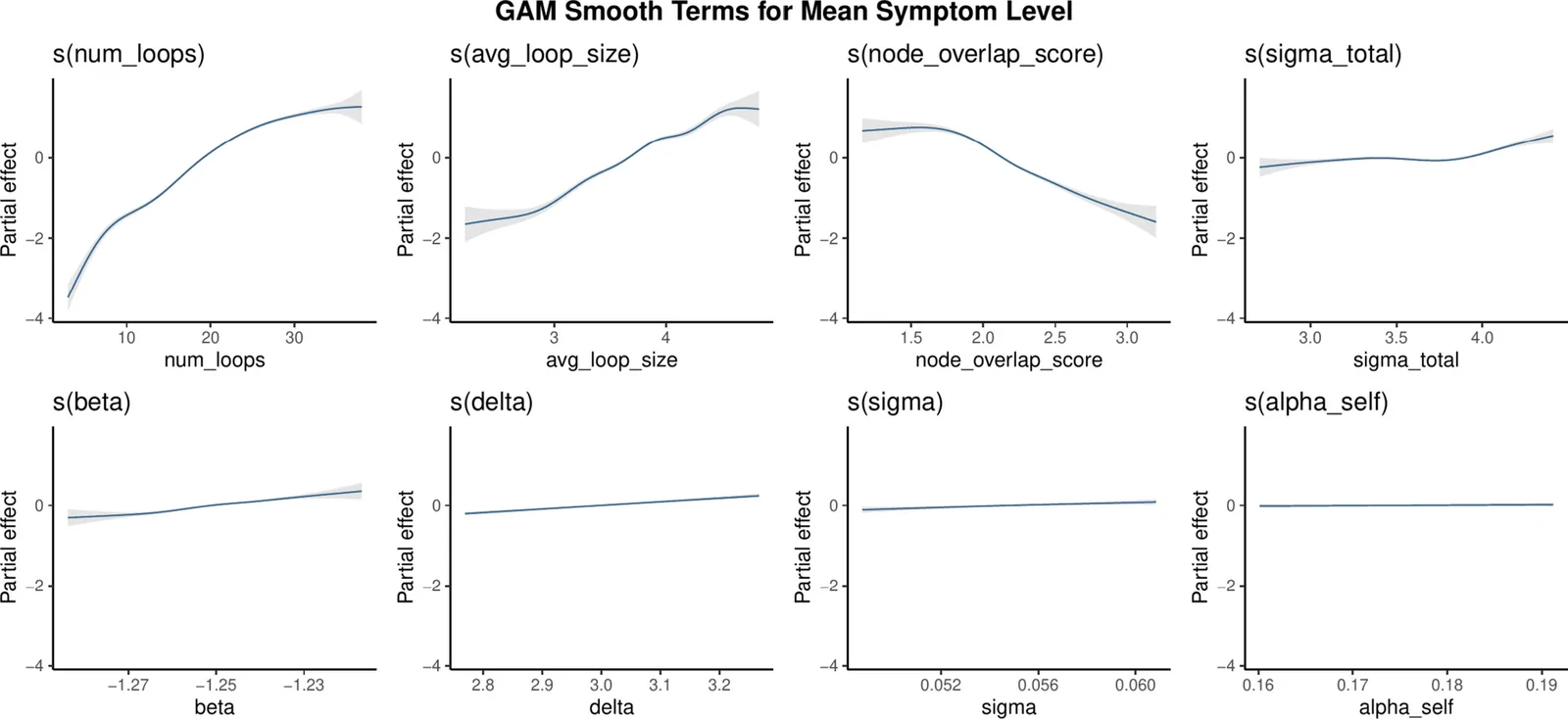
Partial effect plots from a Generalized Additive Model (GAM) predicting mean symptom levels. *Top row*: structural predictors; *bottom row*: average model parameters across 30 simulations. Each panel shows the estimated smooth term and 95% confidence band, controlling for all other variables in the model. Structural metrics show pronounced nonlinear effects (e.g., saturation of loop count, decreasing node overlap), whereas parameter effects are essentially flat

Figure 13 shows the estimated partial effects from the GAM predicting mean symptom levels. Each panel plots the effect of a single predictor while holding all other variables constant—that is, the partial contribution of that predictor to the outcome. This allows us to isolate the unique influence of each structural or parametric feature on symptom activation, even in the presence of correlations among predictors.

In the top row, several structural metrics display clear nonlinear relationships. Feedback loop count is positively associated with symptom burden but shows a saturation effect beyond approximately 25 loops. Average loop size also has a positive association with symptom levels, suggesting that broader feedback structures may reinforce system activation. In contrast, node overlap exhibits a decreasing trend—networks with highly overlapping feedback structures tend to show lower overall activation. This could reflect a diffusion or redundancy of influence in densely looped regions. Degree variability (sigma_total) shows a modest increase, suggesting that asymmetries in node connectivity may contribute to symptom differentiation or persistence. The bottom row shows the effects of the simulation parameters used across runs. These curves are largely flat and centered near zero, suggesting that within the sampled parameter ranges, variation in inputs such as β, σ, and ${\alpha}_{self}$ has comparatively little influence on symptom level, at least relative to differences in structural architecture.

This analysis concludes the full modeling pipeline offered by causalnet—from estimating a skeleton and imposing directional constraints, to generating admissible directed networks, analyzing their topological features, and finally simulating and interpreting their dynamic consequences.

## Conclusion

This tutorial introduced the causalnet package for generating, analyzing, and simulating directed psychological networks. The package provides an end-to-end workflow that begins with an undirected or partially directed skeleton and culminates in dynamic simulations using either user-specified or sampled parameters. By enumerating (all) admissible causal structures, computing topological metrics, and simulating system dynamics under (non)linear models, researchers can systematically investigate how network architecture shapes behavioral trajectories and assess whether architecture-level properties (e.g., feedback concentration) explain dynamics beyond what is attributable to any single edge.

Building on widely used estimation tools such as partial correlation networks and vector autoregressive (VAR/GVAR) models (Epskamp 2020; Epskamp et al., 2018a, b), causalnet extends the modeling pipeline by enabling researchers to incorporate structural uncertainty and simulate across ensembles of admissible directed networks. This allows users to examine how variations in causal configuration, such as the presence or distribution of feedback loops, affect system-level properties such as persistence, recovery, or heterogeneity.

Although our applied example focused on the dynamics of symptoms in depression, the framework is broadly applicable across the domains of psychology and behavioral science. The approach can support a variety of use cases, including exploratory causal discovery, stress reactivity analysis, parameter sensitivity testing, and formal hypothesis evaluation. For example, researchers can compare theoretical causal models by encoding them as constraint matrices and examining their dynamic consequences - providing an empirical basis for model comparison and refinement.

Beyond its immediate analytic utility, causalnet also contributes to the larger project of theoretical development in psychology. As noted previously, the advancement of psychological science requires tools that allow for explicit, dynamic, and testable models (Muthukrishna & Henrich, 2019; Oberauer & Lewandowsky, 2019). By making the causal structure a manipulable object of inquiry, the causalnet framework encourages researchers to move beyond static associations and toward dynamic theories grounded in underlying mechanisms. This flexibility enables new forms of theory generation and falsification, where models are not only specified but simulated, perturbed, and evaluated against the patterns they produce.

In this way, causalnet helps bridge the gap between statistical modeling and psychological theorizing, offering a computational sandbox for exploring how the organization of psychological systems gives rise to complex, time-evolving behaviors. We hope it supports new lines of inquiry into the mechanisms that shape psychological processes and ultimately contributes to more dynamic, testable, and theory-informed models of the mind.

## Supplementary Information

Below is the link to the electronic supplementary material.

Supplementary file1 (DOCX 175 KB)

## Data Availability

All code and materials for the analyses and simulations are publicly available via the causalnet R package on GitHub (https://github.com/KyuriP/causalnet), which is archived on Zenodo at https://doi.org/10.5281/zenodo.17227051. The empirical data used in the applied example (Schramm et al. 2017; Schumacher et al. 2023) are originally hosted by Schumacher et al. (2023) on the Open Science Framework (OSF): https://osf.io/fhqmk/files/osfstorage. For reproducibility, we also include it in the GitHub repository (outside the package distributed via CRAN), together with the OSF source link and the applicable license/citation information (CC BY-NC-SA 4.0).

## Footnotes

1 For example, statistical dependence between two variables may arise through conditioning, indirect pathways, or shared causes, rather than through a direct causal edge connecting those variables (Ryan et al., 2022).

2 For details, see the mgcv package documentation.

3 Each call to s() specifies that the effect of the corresponding variable should be estimated as a smooth (nonlinear) function rather than a linear coefficient. The model remains additive by default, meaning that each smooth term contributes independently to the predicted outcome.

## Code availability

The causalnet R package is openly available on CRAN: https://cran.r-project.org/web/packages/causalnet/. The development version and all tutorial code are available at https://github.com/KyuriP/causalnet.
