# Supplementary material for: A tutorial on causal network simulation and exploration using the causalnet R package
Source: Behav Res Methods. 2026 Jun 22;58(8):204. doi: 10.3758/s13428-026-03030-z (PMC13287159; doi:10.3758/s13428-026-03030-z)
Supplement: Supplementary file 1 — Supplementary file1 (DOCX 175 KB) [file 13428_2026_3030_MOESM1_ESM.docx]

# **Custom simulation models via model_fn**

The simulation engine in causalnet is intentionally designed to allow users to modify the process model (state-update rule) used in dynamic simulations. In addition to the built-in nonlinear and linear models (model_type = "nonlinear" or "linear"), users may supply a custom update function via model_fn (with model_type = NULL). At each time step, simulate_dynamics() computes the network-driven input to each node (the vector interaction) from the directed adjacency matrix and the current state vector current. It then calls the user-defined function model_fn(current, interaction, dt, ...) and expects a numeric vector dS of length $n$ representing per-node increments. The simulator applies the update as next_state = current + dS, optionally adds an external input via stress_event(), and optionally enforces bounds via boundary (e.g., clamping to an interval).

In this appendix, we provide two worked examples using the same small directed network. We visualize the resulting trajectories using causalnet’s built-in plotting function plot_dynamics() for consistency with the main tutorial.

## **Example A: Discrete-time logistic/Bernoulli updates (binary states)**

Some applications naturally treat node states as binary events (e.g., symptom present/absent) at each time step. The following custom model_fn implements a discrete-time logistic/Bernoulli update rule: it computes an activation probability via a logistic link and samples the next state from a Bernoulli distribution. Because simulate_dynamics() applies updates as next_state = current + dS, a custom model_fn must return per-step changes dS. In this example, we first compute next_state via a Bernoulli draw and then return dS = next_state - current.

library(causalnet)

# Example directed network (4-node cycle)
adj_ex <- matrix(c(0,1,0,0,
 0,0,1,0,
 0,0,0,1,
 1,0,0,0), 4, byrow = TRUE)
colnames(adj_ex) <- rownames(adj_ex) <- paste0("Symptom", 1:4)

# Custom discrete-time logistic/Bernoulli model (binary state)
logistic_binary_model <- function(current, interaction, dt, beta, alpha_self) {
 eta <- beta + alpha_self * current + interaction
 p <- 1 / (1 + exp(-eta)) # logistic link
 next_state <- rbinom(length(current), 1, p)
 next_state - current # return increments dS
}

set.seed(123)
params_bin <- list(
 beta = rep(-3.0, nrow(adj_ex)),
 alpha_self = rep(0.7, nrow(adj_ex))
)

S_bin <- simulate_dynamics(
 adj_matrix = adj_ex,
 params = params_bin,
 model_type = NULL,
 model_fn = logistic_binary_model,
 boundary = "none",
 t_max = 200,
 dt = 1,
 S0 = rep(0, nrow(adj_ex))
)

p_bin <- plot_dynamics(
 S = S_bin,
 title = "Custom model: Logistic/Bernoulli (binary dynamics)",
 line_width = 0.5,
 line_alpha = 0.8
)


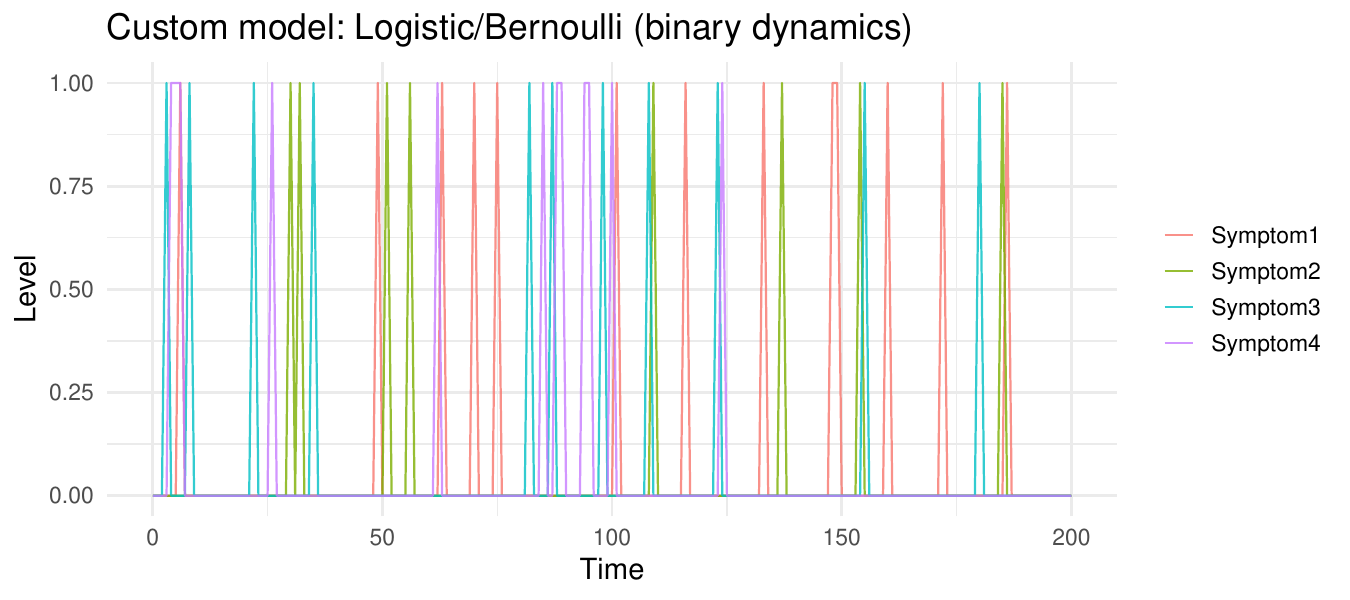


# Figure 14

Worked example of a custom model_fn implementing a discrete-time logistic/Bernoulli update rule. Each node switches between 0 and 1 across time steps, where state changes reflect Bernoulli draws whose probabilities depend on baseline input, self-dependence, and network-driven input.

##

## **Example B: Continuous thresholded and saturating dynamics**

Other domains may prefer continuous-valued states with domain-specific nonlinearities. The next example defines a continuous update rule with three components: (i) thresholding of network input, (ii) saturating growth toward an upper bound, and (iii) additive Gaussian noise. For illustration purposes, we clamp the simulated states to $\left[ 0,1 \right]$ (i.e., values below 0 are set to 0 and values above 1 are set to 1 after each update). We also include an optional, time-localized perturbation via stress_event to show how exogenous inputs can be combined with a custom model_fn.

# Custom continuous model: threshold + saturation + noise
threshold_saturating_model <- function(current, interaction, dt, beta, gain, threshold, sigma) {
 drive <- pmax(0, interaction - threshold) # threshold network input
 dS <- dt * (beta + gain * drive) * (1 - current) # saturating growth toward 1
 dS <- dS + sigma * sqrt(dt) * rnorm(length(current))
 dS
}

set.seed(123)
params_thr <- list(
 beta = rep(0.0, nrow(adj_ex)),
 gain = rep(1.2, nrow(adj_ex)),
 threshold = rep(0.2, nrow(adj_ex)),
 sigma = rep(0.05, nrow(adj_ex))
)

# Optional exogenous perturbation (brief shock)
stress_event <- function(t, state) {
 if (t >= 20 && t <= 30) rep(0.03, length(state)) else rep(0, length(state))
}

S_thr <- simulate_dynamics(
 adj_matrix = adj_ex,
 params = params_thr,
 model_type = NULL,
 model_fn = threshold_saturating_model,
 stress_event = stress_event,
 boundary = "clamp",
 clamp = c(0, 1),
 t_max = 100,
 dt = 0.1,
 S0 = rep(0.01, nrow(adj_ex))
)

p_thr <- plot_dynamics(
 S = S_thr,
 stress_windows = list(c(20, 30)),
 title = "Custom model: Thresholded, saturating continuous dynamics",
 line_width = 0.5,
 line_alpha = 0.8
)


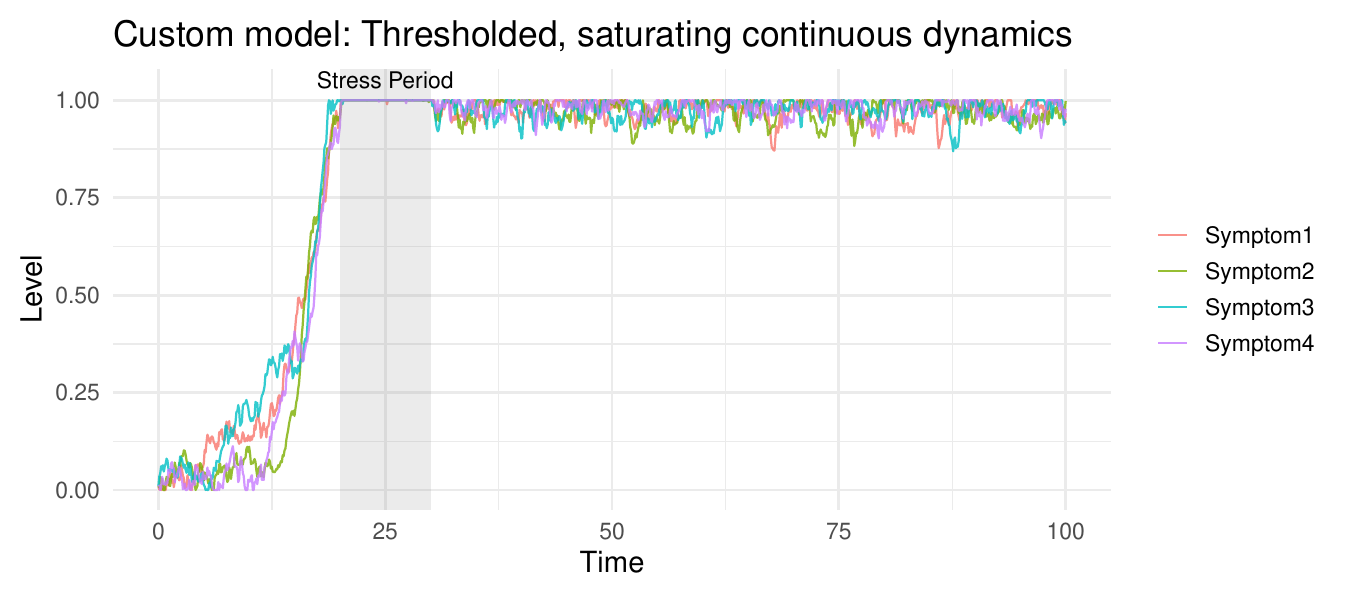


# Figure 15

Worked example of a custom model_fn implementing continuous thresholded and saturating dynamics with additive noise. The shaded region indicates the exogenous perturbation window specified by stress_event; simulated states are clamped to $\left[ 0,1 \right]$ after each update.

These examples illustrate how model_fn can be used to adapt the process model to different substantive assumptions and measurement scales while keeping the same network structure and simulation workflow.
